# Soil microbiome feedbacks during disturbance-driven forest ecosystem conversion

**DOI:** 10.1093/ismejo/wrae047

**Published:** 2024-03-19

**Authors:** Amelia R Nelson, Timothy S Fegel, Robert E Danczak, Marcos V Caiafa, Holly K Roth, Oliver I Dunn, Cosette A Turvold, Thomas Borch, Sydney I Glassman, Rebecca T Barnes, Charles C Rhoades, Michael J Wilkins

**Affiliations:** Department of Soil and Crop Sciences, Colorado State University, Fort Collins, CO 80523, United States; Rocky Mountain Research Station, US Forest Service, Fort Collins, CO 80526, United States; Division of Biological Sciences, Pacific Northwest National Laboratory, Richland, WA 99354, United States; Department of Microbiology and Plant Pathology, University of California Riverside, Riverside, CA 92521, United States; Department of Chemistry, Colorado State University, Fort Collins, CO 80523, United States; The Environmental Studies Program, Colorado College, Colorado Springs, CO 80946, United States; The Environmental Studies Program, Colorado College, Colorado Springs, CO 80946, United States; Department of Soil and Crop Sciences, Colorado State University, Fort Collins, CO 80523, United States; Department of Chemistry, Colorado State University, Fort Collins, CO 80523, United States; Department of Civil and Environmental Engineering, Colorado State University, Fort Collins, CO 80523, United States; Department of Microbiology and Plant Pathology, University of California Riverside, Riverside, CA 92521, United States; The Environmental Studies Program, Colorado College, Colorado Springs, CO 80946, United States; Rocky Mountain Research Station, US Forest Service, Fort Collins, CO 80526, United States; Department of Soil and Crop Sciences, Colorado State University, Fort Collins, CO 80523, United States

**Keywords:** resilience, ecosystem conversion, soil microbiome, metagenomics

## Abstract

Disturbances cause rapid changes to forests, with different disturbance types and severities creating unique ecosystem trajectories that can impact the underlying soil microbiome. Pile burning—the combustion of logging residue on the forest floor—is a common fuel reduction practice that can have impacts on forest soils analogous to those following high-severity wildfire. Further, pile burning following clear-cut harvesting can create persistent openings dominated by nonwoody plants surrounded by dense regenerating conifer forest. A paired 60-year chronosequence of burn scar openings and surrounding regenerating forest after clear-cut harvesting provides a unique opportunity to assess whether belowground microbial processes mirror aboveground vegetation during disturbance-induced ecosystem shifts. Soil ectomycorrhizal fungal diversity was reduced the first decade after pile burning, which could explain poor tree seedling establishment and subsequent persistence of herbaceous species within the openings. Fine-scale changes in the soil microbiome mirrored aboveground shifts in vegetation, with short-term changes to microbial carbon cycling functions resembling a postfire microbiome (e.g. enrichment of aromatic degradation genes) and respiration in burn scars decoupled from substrate quantity and quality. Broadly, however, soil microbiome composition and function within burn scar soils converged with that of the surrounding regenerating forest six decades after the disturbances, indicating potential microbial resilience that was disconnected from aboveground vegetation shifts. This work begins to unravel the belowground microbial processes that underlie disturbance-induced ecosystem changes, which are increasing in frequency tied to climate change.

## Introduction

Disturbances, such as wildfire or logging, are common factors that shape forests and leave legacies that can alter the trajectory of postdisturbance recovery. The subalpine forests of the southern Rockies (USA) are shaped by infrequent stand-replacing wildfire [1] that can release seed stored in cones of lodgepole pine, create seedbeds that favor tree and herbaceous seedling regeneration, reduce surface fuel loads, and alter carbon (C) and nitrogen (N) cycling [2, 3]. Although these ecosystems are adapted to wildfire, climate changes coupled with shifting land use patterns have increased the frequency and severity of wildfires in the western USA [4–6]. Further, the combination of wildfire and other disturbances (e.g. windthrow, drought, or bark beetle infestation) may hamper successful tree regrowth [7–9], which can result in the replacement of forests with nonforest vegetation [10]. Such forest conversions have been documented in response to shifting wildfire activity and compound disturbance, especially near vegetation ecotone boundaries and in arid landscapes [11–14].

The microbial communities (bacteria, archaea, and fungi) that inhabit forest soils drive important biogeochemical processes that influence aboveground forest productivity [15] by providing N and P [16] via mycorrhizal relationships, governing organic matter transformation and C storage [17, 18], and regulating plant diversity [19]. Following a disturbance such as wildfire, changes in the soil microbial community composition or function may directly influence ecosystem processes [20] and could modulate the resistance or resilience of a postdisturbance aboveground forest ecosystem [21].

One such disturbance, pile burning, is the combustion of logging residue on the forest floor, and it is a common management practice to reduce surface fuels that can have impacts on forest soils analogous to those following severe wildfire [22, 23]. The extreme soil heating (>300°C @ 5 cm depth) [24] and duration of smoldering combustion caused by pile burning [24, 25] can result in long-lasting (years to decades) increases in soil pH and nutrient availability [26], loss of soil C, and depleted microbial biomass [24]. Prior research on a five-decade chronosequence of burn pile scars revealed that pile burning could create persistent openings in the forest with a lack of pine tree regeneration and high cover of graminoids (e.g. grasses) and forbs (e.g. *Achillea millefolium*) in contrast to surrounding forest that was successfully regenerating after clear-cut harvesting [27]. Here, we leverage this multidecadal chronosequence of paired burn pile scars and surrounding regenerating forest to investigate the belowground microbial dynamics during divergent ecosystem recovery trajectories.

To identify the soil microbiome processes that underlie this multidecadal burning-induced vegetation-type conversion, we interrogated the soil bacterial and fungal communities using molecular approaches (16S rRNA gene, ITS amplicon, and metagenomic sequencing) to characterize the composition and functional potential of the soil microbiome within the aforementioned series of burn scars along a five-decade chronosequence along with soils collected from the surrounding regenerating forest and parallel pine seedling bioassay *in situ* and greenhouse experiments. This work advances our understanding of the interplay between aboveground vegetation and belowground microbial processes that underpin ecosystem shifts following disturbances, which are predicted to increase in response to climate change. We broadly hypothesized that (i) burn scar soil microbial communities will diverge compositionally over the chronosequence as vegetation shifts over time; (ii) differences in aboveground communities and plant inputs will result in an altered soil microbiome function as related to C and N cycling; and (iii) microbial traits associated with the legacy of fire in burn pile scars (e.g. genes for degrading pyrogenic C) will recede with time and be replaced by traits associated with herbaceous ecosystems.

## Materials and methods

### Field campaign

A chronosequence of burn pile scars that represented pile burning conducted in five separate decades (1960s, 1970s, 1980s, 1990s, and 2000s) were sampled on 20 and 21 July 2020, and these are a subset of burn pile scars utilized in previous studies [26, 27]. This chronosequence of burn pile scars represents pile burns ranging from two to six decades postburning. The burn pile scars were located in northern Colorado on USFS land within the Medicine Bow-Routt National Forest. Lodgepole pine (*Pinus contorta*) is the dominant tree species in the area in relatively even-aged stands. The most abundant soil types are loamy-skeletal, Typic Cryoboralfs and sandy-skeletal, Typic Cryochrepts, and these are formed in sandstone, siltstone, and conglomerate residuum and colluvium and are moderate deep and well-drained to excessively well-drained. See previous studies for an in-depth site description and explanation of site selection [26, 27]. Briefly, USFS stand activity records, which extend back to the 1960s for the Medicine Bow-Routt National Forest, were used to locate harvest units where pile burn operations had been conducted. Selected pile burn openings were identified on aerial photographs and sites were limited to clear-cut, even-age lodgepole pine stands and piles that were burned following clear-cut, were not rehabilitated by the USFS, used a similar amount of fuel, and were roughly the same size (~10–15 m diameter). Additionally, site selection was limited to piles made within harvest units as opposed to larger burn piles created on logging decks where the large pile size and soil compaction may change the impact of burning. Trees with open cones were found in regenerating forests across all sites. The final selected chronosequence of burn pile scars represented pile burning that occurred over five decades from the 1960s to the 2000s. For each decade (60s, 70s, 80s, 90s, and 2000s), there were three units with three piles each (*n* = 9 piles per decade), where we collected depth-resolved (0–10 and 10–15 cm) bulk soil samples (*n* = 18 bulk soil samples per decade; Table S1). At one pile per unit, we also collected depth-resolved samples from just outside the burn scar, which represented regenerating forest after clear-cut that happened at the same time pile burning occurred (e.g. 1960s regen forest sample was clear-cut in the 1960s). Each soil sample was collected with a 10-cm bulb corer and were cleaned with ethanol between samples after brushing away surficial litter and duff. Samples were immediately placed on ice and transported back to the laboratory at Colorado State University (CSU). Soils for DNA extractions were stored at −80°C in the laboratory until processing.

We additionally collected rhizosphere material from lodgepole pine seedlings that were planted within burn pile scars in 2017 for a previous study [26]. We collected rhizosphere material from one seedling within each pile (*n* = 9 per decade) to understand how different times since burn impacts rhizosphere microbiome recruitment and development. To collect rhizosphere material, we dug up the seedling, shook off loose soil not attached to roots, and sampled only soil directly connected to the root system. Samples were immediately placed on ice and transported back to the laboratory at CSU and stored at −80°C in the laboratory until processing. A total of 154 bulk soil and rhizosphere samples were collected (Supplementary Data 1; Table S1).

Because of outlier vegetation (i.e. more dense grass and fewer forbs and woody plants), for all analyses presented herein, we have removed one unit from the 90s (Unit 26) so that the 90s have only 6 rhizosphere samples, 4 control samples, and 12 burned soil samples, plus 8 of these samples utilized for metagenomic sequencing (Table S1). In total, there were 84 burn scar, 28 regen forest, and 42 rhizosphere samples utilized for marker gene sequencing and 28 burn scar and regen forest samples utilized for metagenomic sequencing.

### Soil chemistry

We evaluated soil nutrients and chemistry to gage changes caused by burning and shifting vegetation and to consider the implications of these changes on microbial communities. A subset of 60 bulk soil samples (30 burned and 30 unburned), which included 1 pile per unit per decade, with both burned and unburned shallow and deep samples (*n* = 12 samples per decade, 4 per unit), were selected for chemistry analyses. We analyzed the NO_3_–N and NH_4_–N and dissolved organic C (DOC) and total dissolved N (TDN) released during warm water extracts [28] using ion chromatography (NH_4_-N and NO_3_-N; Thermo Fisher Corporation, Waltham, MA) and TOC-VCPN total organic carbon analyzer (DOC and TDN; Shimadzu Corporation, Columbia, MD, USA). Soil pH was analyzed in a 1:1 soil to deionized water slurry after 1 h of agitation [29] using a temperature-corrected glass electrode (Hach Scientific, Loveland, CO, USA). A set of soils were sieved (2 mm) and dried for 48 h at 60°C and were analyzed for total C and N by dry combustion on a LECO 1000 CHN analyzer (LECO Corporation, St. Joseph, MI, USA). C:N ratios were calculated with %C and %N from the LECO 1000 CHN analyzer data. Soil chemistry data are included in Supplementary Data 1 and, for all analyses, depths were combined to represent the bulk soil chemistry.

### Aerobic metabolism bioassays

Organic matter bioavailability in the soils was determined via laboratory incubations and measuring the production of CO_2_ in sealed bottles over time. Soil incubation experiments were performed in October and November 2020 at Colorado College from soils collected from burn piles ~2 weeks after the primary field campaign explained above. To measure soil organic matter (SOM) bioavailability via soil respiration, incubations were done in triplicate for each whole-soil sample. For incubations, ~30 g of unsieved soil, excluding large rocks or roots, was placed in a glass jar (pre-combusted at 500°C for 5 h) and was left open to the atmosphere at room temperature between incubation time points. At 0, 1, 3, 7, and 14 days, airtight lids were placed on each jar for room temperature incubations (~22°C). MilliQ water was added to samples before incubating on Days 1, 3, 7, and 14 to return to original mass (Day 0) and offset moisture losses due to evaporation. After 2–3 h of incubation, 10 ml of gas from the jar’s headspace was analyzed using the SRI-8610C gas chromatograph (GC). Calibration of the GC was performed using 100, 1000, and 10 000 ppm CO_2_ standard gases, and ambient lab air was used to determine the background CO_2_ (i.e. the concentration of CO_2_ in the jar before the lid was closed) for each incubation. To estimate soil moisture, ~20 g was dried at 50–60°C for ~24 h and was reweighed to obtain soil moisture content gravimetrically. Method was adapted from previously published method [30, 31]. Data are included in Supplementary Data 1.

### DNA extraction, 16S rRNA gene, and internal transcribed spacer amplicon sequencing

Total DNA was extracted from all bulk soil and rhizosphere samples (*n* = 154 total; 84 burn scar, 28 regen forest, 42 rhizosphere) using the Zymobiomics Quick-DNA Fecal/Soil Microbe Kits (Zymo Research, CA, USA). 16S rRNA genes in extracted DNA were amplified and sequenced at Argonne National Laboratory on the MiSeq System using 251-bp paired-end reads and the Earth Microbiome Project primers 515F/806R [32], which targets the V4 region of the 16S rRNA gene. To characterize fungal community composition, the DNA was also PCR amplified targeting the first nuclear ribosomal internal transcribed spacer region (ITS) using the primers (ITS1f/internal transcribed spacer region 2 (ITS2), 33) and was sequenced on the MiSeq platform at the Argonne National Laboratory using 251-bp paired-end reads.

We employed the QIIME2 environment [34] (release 2019.10) for read processing and began from the raw 16S rRNA gene and ITS amplicon sequencing reads, which are both deposited and available at NCBI under BioProject #PRJNA682830. DADA2 [35] was used to filter, learn error rates, denoise, and remove chimeras from reads and, following DADA2, the 16S rRNA gene and ITS amplicon sequencing reads retained, on average, 23 226 and 19 585 reads per sample, respectively. Taxonomy was assigned using the QIIME2 scikit-learn classifier trained on the SILVA [36] (release 138) and UNITE [37] (v8.3) databases for bacteria and fungi, respectively, resulting in 45 009 bacterial and 8708 fungal ASVs. We chose not to rarefy to avoid discarding information, but instead, we converted all data to relative abundance for analysis and rarefaction curves were made for both 16S rRNA gene and ITS amplicon sequencing data to assess whether sequencing was sufficient for comparing alpha diversity between samples (Fig. S1). Ecological guilds were assigned to fungal ASVs using FUNGuild [38] (v1.2). In accordance with FUNGuild creator recommendations [38], we only accepted guild assignments classified as “highly probable” or “probable” to avoid possible overinterpretation and did not retain any ASVs classified as multiple guilds.

All statistical analyses and data visualization were performed in the R environment [39] (v3.6.1). To characterize how microbial populations differed across burn severities and soil horizons, we used the vegan [40] (v2.5-7) and phyloseq [41] (v1.28.0) packages. Nonmetric multidimensional scaling (NMDS) was conducted using Bray-Curtis dissimilarities to examine the broad differences between microbial communities. Permutational multivariate analyses of variance (PERMANOVA) [42] was used to assess how bacterial and fungal communities differed across treatment and time (R package vegan [40], function “adonis2”). We tested for homogeneity of dispersion by sample group using PERMDISP [43] (R vegan function “betadisper”). Mean species diversity of each sample (alpha diversity) was calculated based on species abundance and evenness using Shannon’s Index (H). Linear discriminant analysis with a score threshold of 2.0 was used to determine ASVs discriminant for specific conditions [44].

Surface and deeper microbial community compositions did not significantly differ from one another at most timepoints (Fig. S2), so for all analyses presented, we combined “surface” and “deep” soils to represent the bulk burn scar and regen forest mineral soil column. The similar responses across the depth-resolved samples is likely due to the consistent impacts of slash pile burning on surface and deeper soils that have been reported in other studies [24] due to high fuel loads which can cause large increases in temperature (up to ~300°C) even at 10-cm depth [24, 25], which is in contrast to the lower soil temperatures reached in natural wildfires [45].

### Greenhouse bioassay experiments

To assess the diversity of EMF fungi in the spore bank at each plot, we performed *P. contorta* pine seedling bioassays using established methods [46] from soils collected from each burn pile and surrounding unburned regen forest soils. Seedlings were grown in a common ambient temperature glasshouse at the University of California, Riverside (CA, USA), and stratified *P. contorta* seeds were provided by the Forest Service Rocky Mountain Station (Fort Collins, CO, USA). Seeds were surface sterilized with 30% hydrogen peroxide and were then soaked in water for 48 h [47]. Seeds were germinated on a wetted filter paper for 7–10 days. Pine seedlings were planted in 50-ml Cone-tainers (Super “Stubby” Cell Cone-tainer; Stuewe & Sons Inc., Tangent, OR, USA) using a 1:1 ratio of dried native soil and autoclaved coarse yellow sand to improve drainage. Plants were watered every 3 days and were grown in the glasshouse without fertilizer for ~7 months before harvesting. Treatments were randomized among trays during initial planting and trays were further randomized every other week.

In total, 155 seedlings were planted (five decades × three piles × two treatments × five replicates) plus 5 aerial controls, which consisted only of sterilized potting soil (heated 1 h at 123°C). Plants were harvested by removing the whole plant from the containers and rinsing the soils from the roots with water. Roots were inspected under the dissecting microscope and EMF root tips were collected with sterilized forceps. EMF root tips from an individual seedling were combined into a single tube, flash-frozen, and kept at −80°C until processing.

Frozen EMF root tips were lyophilized, and genomic DNA was extracted using a modified version [46] of the QIAGEN DNAesy Blood and Tissue kit (QIAGEN, Germantown, MD, USA). To identify the EMF fungi present in the root tips, amplification of the rDNA ITS2 was done using the primers ITS3-2024F and ITS4-2409R [33]. PCR, library preparation, and NovaSeq PE250 sequencing (Illumina) were performed by Novogene Corporation Inc., and these are deposited and available at NCBI under BioProject #PRJNA682830. Note that only 32 root tips were used for DNA extractions and ITS amplicon sequencing, as only 32 trees had present root nodules and enough DNA extracted for sequencing.

NovaSeq PE250 sequencing data were processed using QIIME2 version 2020.8 [34]. Denoising was done using DADA2 to remove chimeric sequences and low-quality regions and to produce ASVs. Taxonomic assignments were done using Qiime2 Naïve Bayes Blast + and the reference database UNITE version 8.3 for fungi [37]. Sequences not assigned to the Kingdom Fungi were removed from the ASV tables before subsequent analysis. We assigned functional ecological guilds to each fungal ASV using FUNGuild [38]. All greenhouse bioassay data are included in Supplementary Data 1.

### Community ecological modeling

Two null modeling analyses, β-nearest taxon index (βNTI) and Raup-Crick (Bray-Curtis) (RC_BC_), were performed on the 16S rRNA gene sequencing data to determine how assembly processes governing bacterial community structure differed across treatments and over time [48–50]. A phylogenetic tree was created using the QIIME 2 phylogeny plugin’s “align_to_tree_mafft_fasttree” action. The β-mean nearest taxon distance (βMNTD) was calculated for each possible pairwise sample comparison to find underlying phylogenic contributions to community structure. Using 999 community randomizations to generate a null distribution of βMNTD values, βNTI was calculated to observe the deviation of the observed βMNTD values from the null βMNTD values (“comdistnt,” “picante” R package v1.8). If the resulting |βNTI| was >2, deterministic processes drive community assembly. Communities with a βNTI >2 are more different than would be expected by random chance due to variable selection, whereas communities with a βNTI < −2 are more similar than would be expected by random chance due to homogenizing selection. Differentiating between these processes allows insight into how environmental conditions (e.g. recovery time since burning and vegetation shift) influence the phylogenetic turnover of soil bacterial communities.

If the |βNTI| is <2, communities are as different as expected by random chance and stochastic processes dictate community structure. These stochastic processes can be distinguished using RC_BC_ analyses as dispersal limitation (RC_BC_ > 0.95) where there is a decreased ability for communities to mix, and there is homogenizing dispersal (RC_BC <_ −0.95) where a system experiences high exchange rates. If |RC_BC_| is <0.95, there is no single assembly process strong enough to control community structure and an undominated signal is observed. Because RC_BC_ values are only useful when βNTI indicates stochastic processes, RC_BC_ values are only presented when |βNTI| is < 2. Here, RC_BC_ was calculated according to Stegen *et al*. [51]. Briefly, we used 9999 iterations per pairwise comparison and probabilistically generated null communities based upon microbial abundances from the 16S rRNA gene sequencing data. From these null communities, a null distribution of Bray-Curtis values was calculated and compared to observed Bray-Curtis values and the resulting deviation of the observed values from the null values was normalized from 1 to −1 to calculate the final RC_BC_ value.

For all ecological modeling analyses, the ASV table was rarefied to 15 000 counts due to difficulties processing a feature table with >20 000 ASVs with the “cophenetic” command in the picante R package [52]. R code utilized in Danczak *et al*. (2020) was used here, and it can be found at https://github.com/danczakre/ShaleViralEcology [53]. Note that these analyses were only conducted on the 16S rRNA gene sequencing data because ITS amplicon sequencing data lack the resolution for these analyses. All ecological modeling analyses are presented in Supplementary Data 2.

### Metagenomic assembly, annotation, and binning

A subset of 56 bulk soil samples (28 burn scar and 28 regen forest) were selected for metagenomic sequencing to analyze how microbiome functional potential shifts with recovery postburn. These 56 samples included 1 pile per unit per decade, with both burn and unburned shallow and deep samples (*n* = 12 samples per decade, 4 per unit). To maximize funding utilization, 46 samples were sequenced at the Joint Genome Institute (JGI) and 10 were sequenced at the Genomics Shared Resource, Colorado Cancer Center, Denver, CO. At CU-Denver, libraries were prepared using the Tecan Ovation Ultralow System V2 and were sequenced on the NovaSeq 6000 (Illumnia) platform on an S4 flow cell using 151 bp paired-end reads. For samples sequenced at JGI, an Illumina library was constructed and sequenced 2 × 151 using the NovaSeq S4 platform (Illumina). Sequencing depth ranged from 14 to 25 Gbp from the Colorado Cancer Center and from 22 to 77 Gbp from JGI (Supplementary Data 3). Sequencing adapter sequences were removed from raw reads using BBduk (https://jgi.doe.gov/data-and-tools/bbtools/bb-tools-user-guide/bbduk-guide/) and low-quality reads were trimmed with Sickle [54] (v1.33) with default settings (trimming reads from 5′ to 3′ end, removing reads <20 bp and/or with average quality score < 20). For each sample, trimmed reads were assembled into contiguous sequences (contigs) using the *de novo* de Bruijn assembler MEGAHIT v1.2.9 using kmers [55] (minimum kmer of 27, maximum kmer of 127 with step of 10). Genes were predicted from contigs >2500 bp using Prodigal [56] (ref) (option “-p meta” for metagenome mode; V2.6.2). Predicted genes were clustered at ≥95% identity with MMseqs2 [57], resulting in a final catalog of 16 683 787 nonredundant genes. Trimmed reads were rarefied to 14 Gbp due to a wide range of sequencing depth (14–77 Gbp) and were mapped to the gene catalog using Bowtie2 [58] (v2.3.5). Gene coverage across samples was calculated using coverM contig (v0.6.0; https://github.com/wwood/CoverM) with the “Trimmed Mean” (hereafter, referred to as TMM) method, retaining only those mappings with minimum percent identity of 95% and minimum alignment length of 75%. Genes were annotated using DRAM [59] (v1.4.0). In addition to the DRAM annotations, HMMER [60] against Kofamscan HMMs [61] (Supplementary Data 4) and HMMs from the CANT-HYD database [62] were also used to further identify genes for catechol and protocatechuate meta- and ortho-cleavage, naphthalene transformations, inorganic N cycling, and aromatic hydrocarbon metabolisms. Maximum community doubling times were calculated from codon usage bias using gRodon [63] (v2.0.0; metagenome mode) and gene coverage data (via Bowtie2, v2.3.5).

Assembled contigs (>2500 bp) were binned using MetaBAT2 [64] with default parameters (v2.12.1). Metagenome-assembled genome (MAG) quality was estimated using checkM [65] (v1.1.2) and taxonomy was assigned using GTDB-Tk [66] (v2.1.1). MAGs from all metagenomes were dereplicated using dRep [67] (default parameters, v3.0.0) to create a nonredundant MAG dataset. Low-quality MAGs (<50% completion and >10% contamination) were excluded from further analysis [68], resulting in 786 final MAGs (Supplementary Data 3).

## Results and discussion

### Long-term shifts in aboveground vegetation and soil chemistry

Pile burning catalyzed the shift to a herbaceous-dominated plant community, with higher forb and graminoid cover and lower tree density (average of 366 trees ha^−1^ vs. ~3600 trees ha^−1^) [27] in the openings as compared to the surrounding regenerating pine forest. Changes in soil pH, sulfate, and chloride also persisted across most of the chronosequence (up to six decades post disturbance; Fig. S3). Various N species (e.g. NH_4_^+^, NO_3_^−^, and TDN) were elevated in the most recently burned openings due to the combustion of organic matter, reduced plant N uptake, and enrichment of ash-derived ammonium followed by subsequent microbial nitrification [27] Fig. S3). Changes to belowground inputs associated with the shift from forest to herbaceous community combined with altered soil chemistry could influence the structure and function of the soil microbiome [69].

### Soil microbiome compositional shifts reveal influence of pulse and press disturbance within burn scars

**Figure 1:**
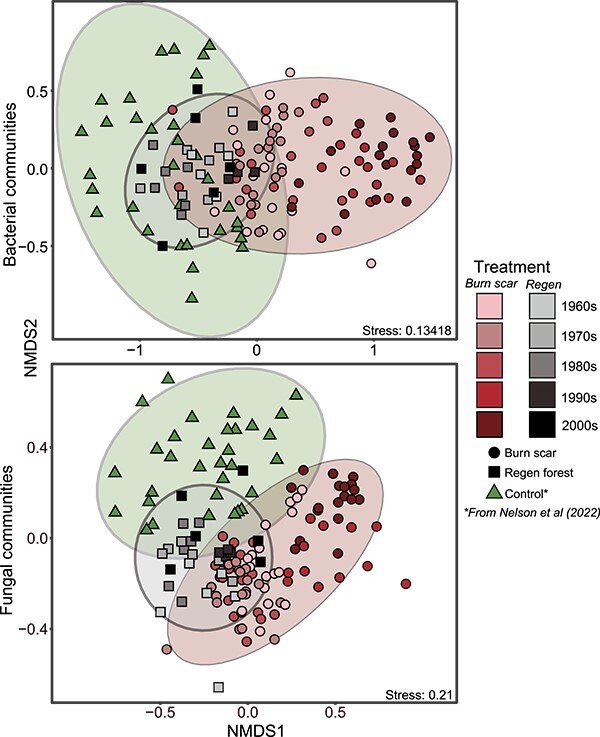
Shifting soil bacterial and fungal community compositions over time since disturbance; NMDS of bacterial (above) and fungal (below) bulk soil communities shows separation of burn scar and regen forest communities that decrease over time; sequencing data from a recent study conducted in a nearby uncut, unburned lodgepole pine forest, were included here as representative control soil bacterial and fungal communities [20]; ellipses show ninety-five confidence intervals around each treatment.

To track soil microbiome changes during ecosystem recovery along distinct ecological trajectories (i.e. postclear-cut forest regeneration and forest shift to herbaceous plant-dominance), we sequenced 16S rRNA genes and ITS amplicons from soil samples (0–15 cm depth) collected in burn pile scars that were burned over five decades (1960s–2000s; hereafter, referred to as “burn scar”) and from adjacent unburned soils in lodgepole pine forest regenerating after clear-cutting (hereafter, referred to as “regen forest”). Burn scar soil bacterial communities were generally compositionally distinct from regen forest soils across the chronosequence (Fig. 1; PERMANOVA *R*^2^ = 0.085, *P* = .001) with interactive effects with time postdisturbance (PERMANOVA *R*^2^ = 0.062, *P* = .001). The soil bacterial communities in the older regenerating forest soils more closely resembled a nearby old-growth lodgepole pine forest than recently regenerated stands [20] (Fig. 1). Soil fungal communities were also statistically distinct between regen forest and burn scar samples, although less so than bacterial communities (Fig. 1; PERMANOVA *R*^2^ = 0.02, *P* = .001), with similar interactive effects with time postdisturbance and treatment (PERMANOVA *R*^2^ = 0.066, *P* = .001). These patterns shifted with time since fire, with treatment having less of an effect on bacterial community composition over time and fungal communities becoming statistically indistinct after six decades postdisturbance (1960s; Fig. S4A). Broad soil microbiome compositional differences were correlated to soil pH, which remained elevated within the burn scars over the chronosequence (Fig. S4B). Thus, despite differences in aboveground vegetation in burn scars, with lodgepole pine generally replaced by graminoids and forbs [27], the soil microbiome was broadly compositionally indistinct from that of the surrounding regenerating forest six decades postdisturbance.

Despite broad soil microbial community similarities, the burn scar soil microbiome harbored fine-scale compositional shifts that were likely associated with pile burning and the subsequent changes in vegetation. For example, soil communities within more recent burn scars displayed similar compositional traits to postwildfire soils that lessened over time since burn. ASVs associated with the fire-responding Actinobacteriota genera *Arthrobacter* [20, 70–72], *Blastococcus* [20, 71, 73], *Modestobacter* [20], and *Massilia* [74, 75] were all generally higher in relative abundance in burn scars relative to regen forest soil samples, with the relative abundance of both *Blastococcus* and *Modestobacter* decreasing over the chronosequence (i.e. recovery time since burning; Fig. S5). By contrast, sequences affiliated with fire-sensitive *Verrucomicrobiota* [20] were depleted in burn scars relative to regen forest soils but steadily increased over the time series (Fig. S6). Similar to other studies [73, 75], more recently burned scar soils contained *Ascomycota*-dominated fungal communities (Fig. S6) which reverted to *Basidiomycota* dominance two decades postburn. There was also a short-lived (three decade) postburn enrichment of taxa within the fungal phyla *Mortierellomycota* (Fig. S6), which display similar trends in the rhizosphere of aspen saplings colonizing severely burned soils [76].

Over time, some changes in the burn scar soil microbiome mirrored shifts observed in the soil microbiome of forests undergoing conversion to grassland. Crowther *et al.* (2014) [77] characterized how deforestation influenced soil microbial communities across different biomes and found consistent compositional shifts, including a decrease in *Basidiomycota*. In the herbaceous-plant dominated burn scar soils, the relative abundance of *Basidiomycota* remained low compared to the regen forest soils after six decades (Fig. S6). In contrast to persistent low EMF diversity in burn scar soils throughout the chronosequence, regen forest soils experienced temporal increases in EMF diversity concurrent with the reestablishment of lodgepole pine [27], resulting in significant differences in the EMF diversity between regen forest and burn scar soils 50 years following disturbance (Fig. S7). Similar to patterns observed after deforestation [78], the relative abundance of *Cyanobacteria* was elevated by pile burning and remained higher in burn scar soils over the chronosequence (Fig. S6). Together, these compositional data reveal that despite broad compositional convergence between burn scar and regen forest soil microbiomes, there are compositional differences likely initiated by aboveground disturbances; burning caused an enrichment of pyrophilous taxa that, as graminoids and forbs established within the burn scar transitioned to communities with greater compositional similarities to those observed in herbaceous-plant-dominated ecosystems.

### Disturbances govern soil microbiome assembly

To determine how assembly processes governing soil bacterial community structure differed over time and across treatments (i.e. pile burning vs. clear-cutting), two null modeling analyses, βNTI and Raup-Crick (Bray-Curtis) (RC_BC_), were performed. These approaches use randomized community structures to identify whether measured communities are more similar or dissimilar to one another than would be expected by random chance. If |βNTI| is >2, deterministic processes drive the community assembly. Communities with a βNTI >2 are more different than would be expected by random chance due to variable selection, whereas communities with a βNTI < −2 are more similar than would be expected by random chance due to homogenizing selection. These assembly processes are driven by environmental conditions, where a homogenous environment (e.g. spatially or temporally) might result in homogenizing selection and heterogeneous conditions (e.g. variable soil pH) might cause variable selection processes [79]. If |βNTI| is <2, communities are as different as expected by random chance and stochastic processes dictate community structure. These stochastic processes can be distinguished using RC_BC_ analyses as dispersal limitation (RC_BC_ > 0.95) where there is a decreased ability for communities to mix and as homogenizing dispersal (RC_BC <_ −0.95) where a system experiences high exchange rates. If |RC_BC_| is < 0.95, there is no single assembly process strong enough to control the community structure and an undominated signal is observed.

**Figure 2:**
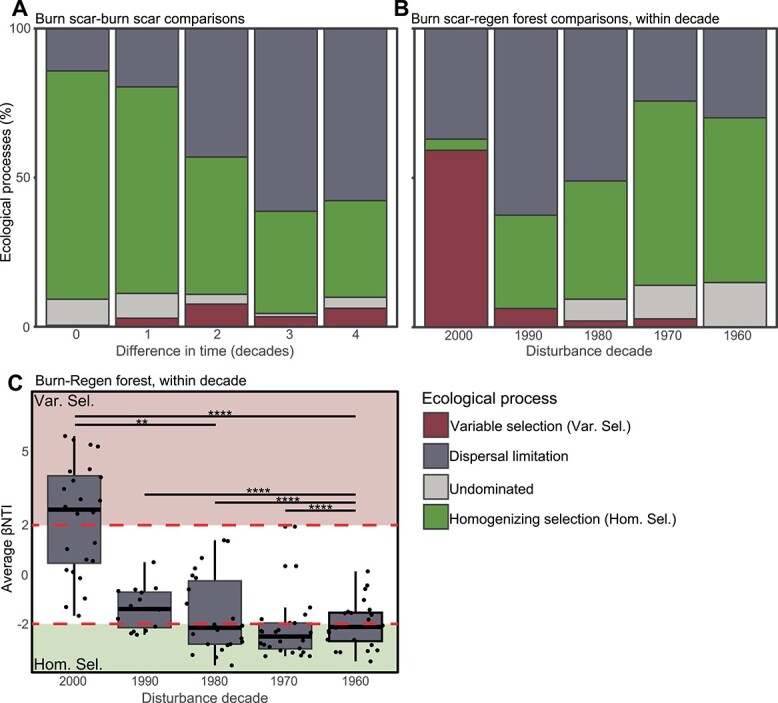
Ecological processes governing bulk soil bacterial community structure differ through time; (A) proportion of assembly processes, derived from βNTI and RC_BC_ analyses, dictating community structure from within-treatment (burn scar) comparisons plotted by difference in time between burn scars (e.g. 2000s–1970s comparison, three-decade difference); these data show that burn scar bacterial communities are more similar to one another when closer in disturbance age; (B) proportion of assembly processes from within-decade comparisons of treatments (e.g. 2000s burn compared to 2000s regen forest), which indicate that the burn scar and regen forest microbiomes are more different to one another with less recovery time postdisturbance; (C) average βNTI from within-decade comparisons of treatments, showing that, over time postdisturbance, burn scar and regen forest microbiomes become more similar to one another than expected by random chance; the lower and upper hinges of the boxplots represent the 25th and 75th percentiles and the middle line is the median; the upper whisker extends to the median plus 1.5× interquartile range and the lower whisker extends to the median minus 1.5× interquartile range; significant differences between burn scar and regen forest samples indicated with asterisks as indicated by Wilcoxon rank-sum test; ^*^P < .05, ^**^P < .01, ^***^P < .001, ^****^P < .0001; note that homogenizing dispersal was not observed in this system.

Combined, βNTI and RC_BC_ results revealed a diminishing impact of pile burning on soil bacterial community assembly over time. Burn scar soil bacterial communities experienced more homogenizing selection (i.e. communities are more like one another than expected by random chance) when they were more similar in age (Fig. 2A). These data suggest that burn scars follow very similar recovery trajectories over time, as within-decade community comparisons (e.g. all 2000s–00s and 1990s–90s) are dominated by homogenizing selection. With greater time between comparisons (e.g. a 1960s–2000s burn comparison vs. a 1960s–60s burn comparison), the influence of both variable selection and dispersal limitation increased. The observed shift in processes governing soil bacterial community structure with time indicates the lessening of fire influence and the role of additional environmental factors (e.g. differing plant carbon soil inputs) in shaping community assembly during ecosystem shift from forest to herbaceous plant-dominated.

Null model analyses between burn scar and regen forest soils also revealed evidence for the decreasing influence of fire on bacterial communities with time. Within-decade treatment comparisons (Fig. 2B and C) highlighted greater variable selection (i.e. communities are more different than one another than expected by random chance) in the 2000s, which greatly decreased over time to be replaced by predominantly homogenizing selection processes. Thus, these data indicate that following the fire pulse disturbance, the resulting press disturbance of altered soil physicochemical conditions and altered aboveground vegetation exerted significant control on soil bacterial community assembly for up to three decades. Beyond this, dispersal limitation and homogenizing selection (Fig. 2B and C) influenced soil bacterial community structure within the two conditions. Therefore, under many situations in burn scar and regen forest soils disturbed between 30 and 60 years ago, the communities experience sufficiently similar environmental conditions to develop homogenously (e.g. subject to homogenizing selection). However, if selection is too weak, these communities proceed to develop due to dispersal limitation due to the lack of strong dispersal capabilities between burn scar and regen locations.

### Distinct ecosystem trajectories alter microbiome function potential for C and N cycling

**Figure 3:**
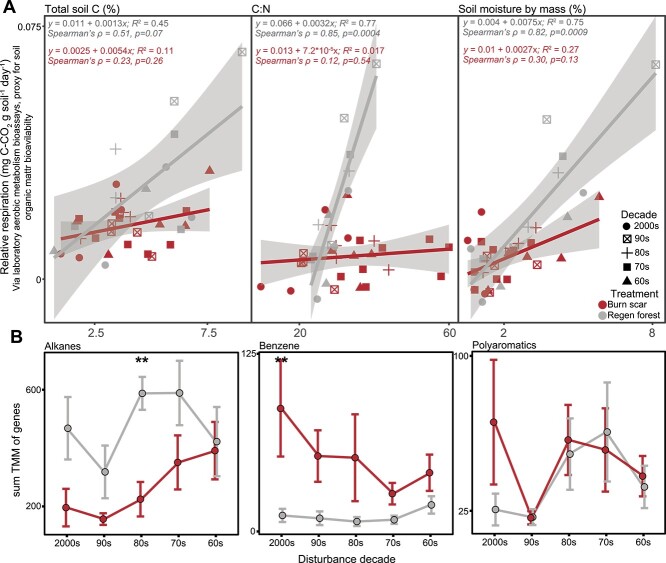
Microbially mediated C cycling influenced by disturbance and recovery trajectories; (A) relative soil respiration calculated from laboratory bioassays relations with total soil C (%), C:N, and soil moisture, with linear regression and Spearman’s correlation statistics; shaded area shows 95% confidence interval of linear model; soil respiration and inferred organic matter bioavailability are significantly positively correlated to all three soil variables in Regen forest soils, but not the burn scar soils; (B) average summed TMM of M-values of genes associated with the bacterial degradation of alkanes, benzene, and PAHs from metagenomes derived from burn scar and regen forest soil samples; significant differences between burn scar and regen forest samples indicated with asterisks as indicated by Wilcoxon rank-sum test; ^*^P < .05, ^**^P < .01.

To identify impacts of ecosystem conversion on soil microbial functional potential for C and N cycling, we leveraged a gene database derived from 56 metagenomes from burn scar and regen forest soils for genes associated with aromatic catabolism (e.g. polyaromatic degradation, b-ketoadipate pathway; *n* = 26 241 genes), the processing of alkanes (*n* = 814), carbohydrate degradation (CAZymes; *n* = 275 398), and the cycling of inorganic N (*n* = 6686). Pile burning and clear-cutting both reduced soluble soil C (i.e. DOC; Fig. S3) which then recovers as herbaceous plant or tree roots proliferate and forest litter inputs increase. Soil DOC concentrations increased over time in both treatments, likely following two different mechanisms: in burn scars, DOC increase may derive from degradation of bulkier, low solubility fire-derived polyaromatic hydrocarbons (PAHs) into smaller water-soluble compounds, and root exudation from quickly establishing grasses. In regen forest soils, DOC increase followed the reestablishment and growth of lodgepole pine through litter and root exudate inputs. Burn scar soils generally had higher DOC, with supporting previous work showing an increased total C in burn scar soils relative to regen forest [27]. However, relative soil respiration and inferred SOM bioavailability were generally lower in burn scars soils relative to regen forest soils (Fig. S8), potentially due to limited pine reestablishment within burn scars and lower C release via root exudation of annuals relative to perennials [80]. As expected, respiration was positively correlated to %C, C:N, and initial soil moisture in regen forest soils, whereas these trends were absent in burn scar soils (Fig. 3A). This suggests that other factors such as microbial access to substrates or the genomic potential to utilize available C may exert a greater influence on soil respiration within the burn scar soils.

To investigate whether the metagenome-encoded potential for utilizing C substrates differed between burn scar and regen forest soils, we quantified the relative abundance of specific genes over time and between treatments. Burning can transform SOM to increasingly aromatic molecular structures [81], like PAHs, which become more aromatic with increased burn severity. Because of the prevalence of aromatic pyrogenic organic matter (pyOM) within burned soils [81, 82] and studies indicating microbial potential for degrading pyOM in burned soils [20], we identified genes targeting both PAHs and monoaromatic compounds (e.g. benzene). We found that genes for bacterial degradation of PAHs and benzene were enriched in the most recent burn scars relative to regen forest soils, likely due to residual pyOM from slash pile burning (Fig. 3B). PAH degradation genes were linked to MAGs that represented the Proteobacteria family *Xanthobacteraceae* (BP_680, BP_689, BP_693, BP_694), the Actinobacteria genus *Mycobacterium* (e.g. BP_237), the Desulfobacterota family *Binataceae* (e.g. BP_616), and the Myxococcota genus *Labilitrix* (BP_638), revealing diverse community members that encode the functional potential for degrading PAHs within burn scar soils. The relative abundance of PAH degradation genes quickly declined 30 years following pile burning (1990s) and continued to mirror gene relative abundance profiles in regen forest soils throughout the remainder of the chronosequence (Fig. 3B). By contrast, genes encoding benzene degradation remained enriched within burned soils through much of the chronosequence, likely because monoaromatic compounds can also be derived from root and plant litter inputs, revealing a longer-term legacy that follows the impact of burning (e.g. altered plant inputs). Combined, these data reveal clear pyrophilous traits encoded by the soil microbiome that persisted for several decades following burning (more detailed in Supplementary text; Fig. S13). In contrast to these trends, genes encoding pathways for the degradation of alkanes (representing more simple aliphatic compounds) were enriched in regen forest soils up to 60 years postdisturbance (1960s soils; Fig. 3B), representing the likely greater concentrations of more simple bioavailable substrates within regen forest soils.

Relative abundance profiles of CAZymes further indicated altered genomic potential in more recently disturbed soils, with burn scar and regen forest soils becoming more similar ~60 years postdisturbance (1960s samples). For example, polysaccharide lyases, which catalyze the decomposition of acidic polysaccharides (e.g. starch and chitin), were enriched in recently burned soils with many differentially abundant genes (via DESeq2, *P* < .05; *n* = 147 genes enriched in 2000s burn scar vs. 81 in regen forest; Fig. S9). By contrast, carbohydrate esterases, which include enzymes involved in hemicellulose and pectin metabolism, displayed opposite trends and were enriched in regen forest soils (Fig. S9). Carbohydrate esterase normalized abundance was significantly correlated with time since disturbance (Spearman’s *ρ* = 1, *P* = .0167; Fig. S9), recovering to resemble regen forest soils by the 1960s. Polysaccharide lyases also recovered to similar normalized abundances as regen forest soils by six decades postdisturbance, suggesting that some substrate pools are equally abundant after six decades postdisturbance. In contrast to conifer forests burned by high-severity wildfire in California [70], where glycoside hydrolases were enriched in burned soils and attributed to C limitations, we identified no clear difference in their relative abundances over time between the two treatments.

Inorganic N was elevated the first decade following pile burning due to combustion and release of NH_4_^+^ from forest biomass and surface organic matter combustion and increased potential for nitrification (Fig. S3). The influx of NH_4_^+^ is associated with a short-term (<10 year) postfire increase in nitrification [70, 83]. We measured higher diversity of putative nitrifiers in burn scar relative to regen forest soils over the chronosequence (Fig. S10B), although genes encoding nitrification (“amoABC”; *n* = 50) were less abundant over the same samples (Fig. S10A). By contrast, denitrification gene profiles (e.g. *nirK*, *nirS*, *narG*, *nosZ*; *n* = 4235) did not differ between burn scar and regen forest soils (Fig. S10), potentially reflecting the broad distribution of this functional trait across diverse bacterial lineages [84]. The only inorganic N function enriched in burned soils was N fixation (*nifH*; *n* = 22) which was elevated in the 2000s burn scars (Fig. S10). These *nif* genes were linked to three MAGs all associated with the Actinobacteria order *Actinomycetia* (BP_140, BP_141, and BP_201), which have been found to play an important role as diazotrophs in desert soils [85]. Mirroring trends observed with C cycling functions, the normalized abundances of genes encoding microbially mediated N cycling (apart from nitrification) converged over time across both treatments.

Together, these data highlight the strong filtering effect of burning, which exerts combined pulse and press disturbances on soils that last multiple decades and are detected through significant differences in the functional potential of the soil microbiome. However, despite divergent ecological trajectories between regen forest and burn scar sites, and long-term differences in vegetation and associated soil carbon inputs, the longer-term convergence in functional potential for C and N cycling indicates the weakening of environmental filtering processes that drive differences in the soil microbiome functions over time.

### Burn scars have lasting impacts on key soil functions

**Figure 4:**
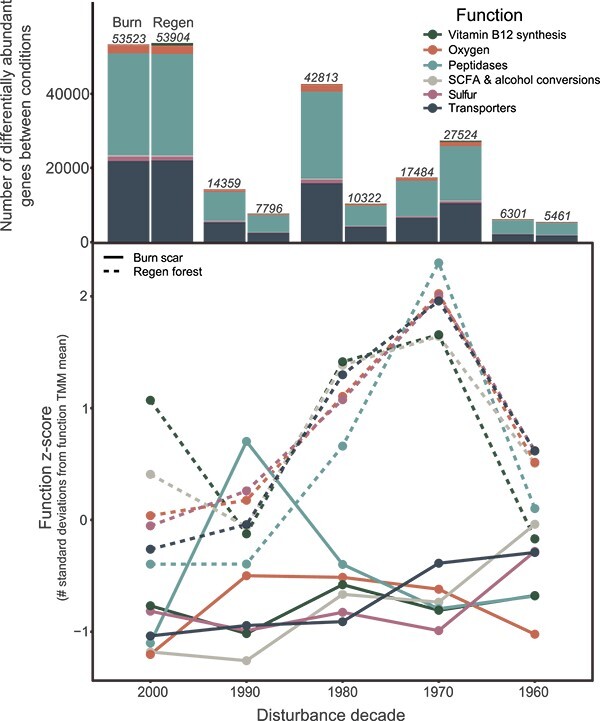
Ecologically relevant functions remain depleted in burn scars despite broad convergence of function over time; the function *z*-score (deviation from function mean across all time points and treatments; derived from function TMM across time between regen forest (dashed line) and burn scar (solid line) soils, calculated within each function; above, the number of differentially abundant genes within these functional categories between the two treatments over time.

Functional potential between the burn scar and regen forest soil microbiomes generally converged over the chronosequence, with the number of differentially abundant genes between the two treatments (via DESeq2 [86]; *P* < .01) decreasing from 433 526 to 80 941 from the 2000s to 1960s (Table S2). However, there were still long-lasting influences of burning on certain ecologically relevant functions encoded within the burned soil microbiome (Fig. S11; Fig. 4). One such process is the biosynthesis of cobalamin (vitamin B_12_, *cob* genes), an important coenzyme involved in gene regulation and the synthesis of nucleotides and amino acids. Cobalamin production is conserved within a relatively small group of microorganisms and serves as a keystone function within ecosystems [87]. Genes encoding the bacterial synthesis of cobalamin from cobinamide displayed greater relative abundances in regen forest soils across the entire time series with no convergence after six decades (Fig. 4). Further, samples from regen forest soils also contained more differentially abundant genes associated with this process (*n* = 13 in 1960s regen forest) that linked to MAGs (via BLAST) affiliated with the Actinobacteria genera *Mycobacterium* (BP_728 and BP_240) and *Pseudonocardia* (BP_325) along with the Proteobacteria genera *Caballeronia* (BP_718) and *Aliidongia* (BP_649). Indeed, all noted genera associated with cobalamin synthesis had lower relative abundances in burn scar soils relative to regen forest soils over the entire chronosequence. Given the reliance of many soil bacteria on exogenous cobalamin [88] and its role as a cofactor in a broad array of bacterial enzymes [87], the trends observed here could influence soil microbiome activity and influence plant recovery within the burn scars [89].

In soils, where proteinaceous compounds are the most abundant form of soil organic N [90], peptidases degrade high molecular weight peptide N to simpler forms (e.g. amino acids) as part of a critical strategy used by microbes to gain bioavailable N in N-limited conditions. The depolymerization of peptide N is additionally considered as a rate-limiting step for terrestrial N cycling, as it increases bioavailable N for both plants and microorganisms [91, 92]. Here, we found greater relative abundances of genes encoding peptidases in regen forest soils relative to burn scar soils in the later decades following disturbance (i.e. 1980s, 1970s, and 1960s; Fig. 4). Although inorganic N chemical profiles converge over the chronosequence (Fig. S3), these data suggest that burn scar soils are more limited by available sources of organic N than regen forest soils six decades following disturbance.

Other broad functions that were more abundant in regen forest soils six decades following the disturbances are general transporters, sulfur (within DRAM summary output, “energy”), short-chain fatty acid and alcohol conversions, and the DRAM header “oxygen,” which mainly includes genes encoding for cytochromes (e.g. *coxA*; cytochrome c oxidase subunit I (EC:1.9.3.1)) (Fig. 4). Increased relative abundances of transporters indicates the prevalence of resource acquisition strategies within the regen forest microbiome and an increased investment in the extracellular enzymatic machinery for resource capture, potentially at the expense of growth yield [93]. Increased relative abundances of genes encoding for electron transport chain cytochromes (*n* = 43 249 genes) might indicate an increased respiratory activity and ATP yield, and the increased relative abundance of genes participating in sulfur cycling—the majority of which are for assimilatory sulfate reduction (20 704 of the 26 627 genes)—could also indicate an increased microbial activity and growth in regen forest soils.

### Altered soil microbiomes may contribute to limited pine establishment following pile burning

Pine seedlings require key soil microbial symbionts in the rhizosphere for optimal survival and growth. Despite the dense regeneration of lodgepole pine surrounding the burn scars, tree colonization is rare within the burn scars and they become graminoid- and forb-dominated over the chronosequence [27]. To investigate the belowground processes that may influence tree regeneration within the burn scar openings, we conducted greenhouse pine bioassay growth experiments with soils collected from the sites and sampled the rhizosphere of *in situ* lodgepole pine seedlings planted within the burn pile scars in summer of 2017 (*n* = 9 per decade). An earlier study on this series of pile burn scars found that seedling survival and EMF colonization was lowest in the most recently burned scars, although overall seedling growth and survival in the burn scars was high [26]. Here, in greenhouse pine seedling bioassay seedling experiments, we observed a lower EMF relative abundance in root nodules on pine seedlings grown in recently burned soils (i.e. 2000s; Fig. S12A) that corroborated the low colonization of EMF found on pine seedling roots in the earlier study [26]. Despite the overall high tree mortality and low root colonization (Supplementary Data 1), the few EMFs that did colonize greenhouse pine seedling root nodules included *Rhizopogon*, *Suillus*, *Cenoccocum*, and *Wilcoxina*, all common spore bank fungi known to persist in postfire soils [46, 94–96] (Fig. S12C). Thus, in spite of the persistence of some well-known EMF, overall, the results suggest that pile burning depletes pine-associated EMF spore banks that are abundant in most soils [46].

**Figure 5:**
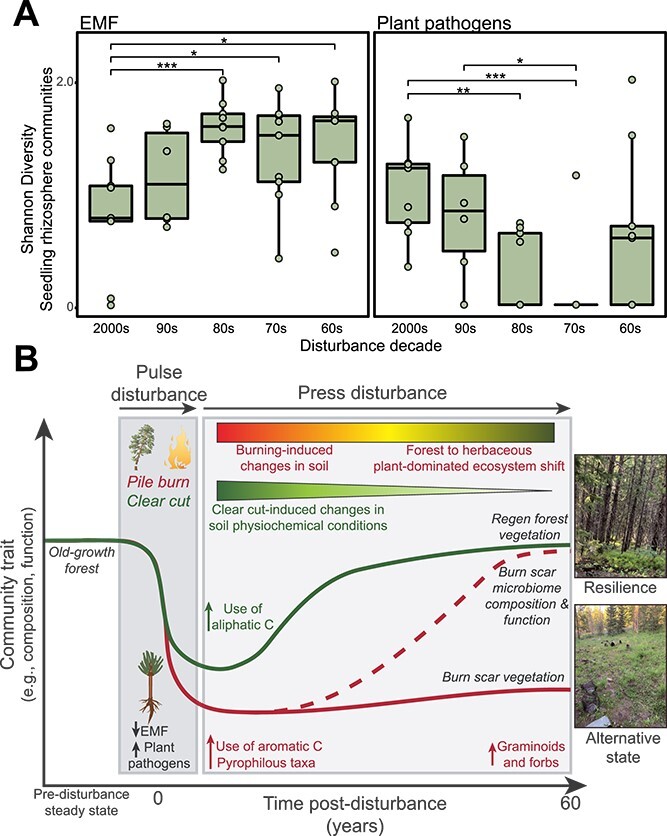
Seedling rhizosphere in recently burned soils depleted in beneficial EMF communities; (A) Shannon index of EMF (left) and plant pathogen (right) communities in rhizosphere of seedlings planted within burn scars; significant differences between burn scar and regen forest samples indicated with asterisks as indicated by Wilcoxon rank-sum test; ^*^P < .05, ^**^P < .05, ^***^P < .01, ^**^^*^P < .0001; (B) conceptual diagram overviewing the ecosystem trajectory following disturbances in burn scar (i.e. pile burning) and regen forest (i.e. clear-cut) soils; both systems experience a pulse disturbance which causes a shift in vegetation and soil microbial communities, after which there is a long-term press disturbance; within regen forest soils, the press disturbance is due to altered soil chemical conditions caused by clear-cut, which lessens over time; by contrast, burn scar soils experience a press disturbance throughout the chronosequence, initially caused by fire-induced changes to soil physiochemical properties, which lessen over time and are replaced by a press disturbance caused by the ecosystem shift; during this ecosystem conversion, the aboveground community shifts from pine- to graminoid- and forb-dominated, but soil microbiome composition and function broadly begin to converge with regen forest soils.

In corresponding *in situ* lodgepole pine seedling rhizosphere samples (i.e. seedlings planted within the burn pile scars), we found significant decreases in the rhizosphere EMF community diversity in pines planted in more recent burn scars (2000s; Fig. 5A). Although some of the aforementioned postfire spore bank fungi—*Rhizopogon*, *Suillus*, and *Wilcoxina* [46, 94–96]—were generally present in the pine seedling rhizosphere across the chronosequence, there was a lack of other known EMF symbionts for lodgepole pine. For example, the Basidiomycota genera *Cortinarius* remained depleted in burn scar soils over the chronosequence (average 0.8% relative abundance), whereas it rebounded in regen forest soils (7.3% relative abundance in 1960s regen forest) and was completely absent in rhizosphere samples. Although lodgepole pine regeneration is typically stimulated by clear-cutting, the combination with pile burning appears to have depleted the local EMF recolonization potential. Further inhibiting seedling success and growth, rhizosphere samples from seedlings planted *in situ* also hosted a significantly higher diversity of plant pathogenic fungi, which was relative to analogous samples from older burn scars (Fig. 5A). Dove *et al.* (2021) [76] reported an increased relative abundance of plant pathogens in the rhizosphere and leaf phyllosphere of aspen saplings in wildfire burn scars just 1 year postfire, and our findings suggest that such effects may persist for extended time periods in soils impacted by high-severity fire. Combined, data from greenhouse pine bioassay experiments and *in situ* lodgepole pine seedings suggest that pile burning has an adverse effect on vital EMF partners for lodgepole pine. A subsequent increase in plant pathogenic fungi might hinder the early success of pine within the burn scars, allowing the persistence of understory species (i.e. graminoids and forbs) and facilitating the conversion to a herbaceous plant-dominated ecosystem (Fig. 5B).

## Conclusion

Within subalpine conifer forests of the Southern Rockies, the increase in compound disturbances can catalyze the conversion from forest to nonforest vegetation. Here, we use pile burns as a surrogate of severe wildfire to investigate changes in the soil microbiome over the course of six decades following a compound disturbance comprised of pile burning following clear-cut harvesting. We used paired comparisons of postfire changes in soil beneath nonforest burn scars and lodgepole pine regrowth following clear-cut harvesting. Initial loss of lodgepole pine EMF partners following burning and increased plant pathogen abundance in addition to other factors (i.e. low seed availability and high seed predation) likely contributed to low tree seedling establishment within the scars. We also report a loss of ecologically relevant microbial functions (e.g. peptidases) that may further inhibit successful seedling reestablishment. Despite these short-term impacts (i.e. two decades), after six decades, the soil microbiome within the burn scars recovered to generally resemble regen forest soils in both composition and function, revealing belowground resilience in response to disturbance-induced ecosystem conversions. The recovery of the soil microbiome described here might be influenced by the small spatial extent of the burn scars (~10 m in diameter) surrounded by closed canopy forest and further analyses of larger scale ecosystem conversions are needed to advance understanding. This unique dataset provides an invaluable insight into the belowground microbial dynamics that underly aboveground ecosystem shifts within these vital and vulnerable terrestrial ecosystems.

## Supplementary Material

Nelsonetal_Supplementary_Information

Nelsonetal_Supplemental_Data_1

Nelsonetal_Supplemental_Data_2

Nelsonetal_Supplemental_Data_3

Nelsonetal_Supplemental_Data_4

## Data Availability

The metagenomic reads, bacterial MAGs, 16S rRNA gene sequencing reads, and ITS amplicon reads reported in this paper have been deposited in National Center for Biotechnology Information (NCBI) BioProject PRJNA682830. NCBI Accession numbers for 16S rRNA gene and ITS amplicon sequencing reads can be found in Supplemental Data 1. NCBI Accession numbers for metagenomes and bacterial MAGs are included in Supplemental Data 3.
