# Supplementary material for: Soil microbiome feedbacks during disturbance-driven forest ecosystem conversion
Source: ISME J. 2024 Mar 19;18(1):wrae047. doi: 10.1093/ismejo/wrae047 (PMC11650388; doi:10.1093/ismejo/wrae047)
Supplement: Nelsonetal_Supplementary_Information [file nelsonetal_supplementary_information.pdf]

Supplementary Information for
Soil microbiome feedbacks during disturbance-driven forest ecosystem conversion

Amelia R. Nelson[1], Timothy S. Fegel[2], Robert E. Danczak[3], Marcos V. Caiafa[4], Holly K. Roth[5], Oliver I. Dunn[6], Cosette A. Turvold[6], Thomas Borch[1,5,7], Sydney I. Glassman[4], Rebecca T. Barnes[6], Charles C. Rhoades[2], Michael J. Wilkins*[1]

Corresponding Author (*):
Dr. Michael J. Wilkins
Colorado State University
Department of Soil and Crop Sciences
1170 Campus Delivery
Fort Collins, CO 80523-1170
Mike.wilkins@colostate.edu

This PDF file includes:

Supplementary Text	2-4
Supplementary Figures 1-13	4-17
Supplementary Table 1-2	18-19
Supplementary Dataset Information	20
References	21

Other supporting materials for this manuscript include the following:

Supplementary Data 1-4

Supplementary text

Fire legacy soil microbiome traits differ in prevalence at longer timescales

Previous studies focusing on the post-fire soil microbiome have identified prevalent traits that allow specific taxa to thrive and become dominant in soils with altered physicochemical characteristics(1–3). Using our extensive gene dataset from 56 metagenomes from both burn scar and regen forest soils across a multidecadal chronosequence, we inventoried three recurring traits to assess their importance within the burn scar soil microbiome over multidecadal timescales. These traits included (1) fast growth rates to enable quick occupation of newly available niche space following disturbance, (2) stress response genes to allow their survival within the altered post-burn soils, and (3) the ability to degrade fire-transformed pyrogenic carbon with increased aromaticity.

Previous studies have found that fast growth potential is a key characteristic that governs soil successional dynamics one year following wildfire(2,3) but that its significance may wane after five years(4). In contrast, another fire chronosequence study performed in the Sierra Nevada found that communities had relatively fast growth rates multiple decades post-fire due to an increase in microbial metabolic substrates(1). Here, we investigated whether fast growth was a dominant trait within the burn scars via estimates of maximum growth rates of bulk soil metagenomes(5). Following the removal of 3 samples that had a maximum growth rate >5 hours, the average maximum growth rates for soil microbiomes within burn scar and regen forest soils were 3.67 and 3.25 hours, respectively. Over the chronosequence, there was no evidence that the burned soil microbiome harbored faster maximum growth rates relative to regen forest soils, indicating that this trait plays a lesser role in structuring microbial communities across decadal timescales post-fire (**Fig. S13**). Here, we suggest that altered vegetative successional dynamics following slash pile burning likely influences the substrates available for microbial growth and may account for observed differences with natural wildfires.

Stress response is another important trait that allows taxa to persist in burned soils that generally have lower moisture due to loss of overstory causing increased temperatures and fire-induced soil hydrophobicity. The genomic potential for biosynthesis

of two such stress protectants, mycothiol and ectoine, is enriched in MAGs in early post-fire soils(1) (i.e., 4 years post-fire). Mycothiol is produced by Actinobacteria for oxidative stress tolerance(6), which is likely high following burning due to increased hydrophobicity and decreased water holding capacity of soils, and ectoine aids in rapid temperature fluctuations and ionizing radiation damage(7). Here, genes for synthesizing ectoine were more abundant in burn scar vs. regen forest soils over nearly the entire chronosequence (**Fig. S13**). The enrichment of ectoine synthesis genes is likely due to the long-term loss of overstory within the burn scars, which remain depleted in shade-producing pine species and become graminoid- and forb-dominated(8). In contrast, genes for mycothiol synthesis were not significantly more abundant in burn scar vs. regen forest soils, revealing that oxidative stress tolerance is no longer an important trait decades following burning. We additionally inventoried the gene dataset for genes necessary for trehalose and glycine betaine synthesis, heat shock, and sporulation, and found that these stress response genes were not consistently enriched in burn scar soils relative to regen forest (**Fig. S13**), indicating that the enrichment of stress response genes with wildfire is no longer prevalent decades following wildfire. Instead, differences in the abundances of these genes here (e.g., ectoine synthesis) are likely due to the divergent ecological trajectories between burn scars and regen forest soils.

Another important trait for the soil microbiome that colonizes and persists in soils following high temperature burning is the ability to utilize pyOM, which is generally increased in aromaticity as compared to unburned soils(9). Briefly, we found that genes associated with the bacterial degradation of PAHs and benzene were enriched in more recently burned scars relative to regen forest soils (**Fig. 3b**). These enriched genes decline by 30 years following pile burning, likely due to decreasing pyOM concentrations over time since burn. Benzene degradation genes also remain enriched within burned soils over much of the chronosequence (**Fig. 3b**). This metagenomic sequencing data reveals that the trait for pyOM utilization in the burned soil microbiome is important within burn pile systems decades following burning.

Combined, these data reveal that key traits governing soil microbial dynamics in the immediate aftermath of fire are either not important over these multidecadal timescales (fast growth rates), differ due to the ecosystem conversion of the burn scars

to herbaceous plant-dominated ecosystems (stress response), or show waning importance over time since burn (pyrogenic C utilization).

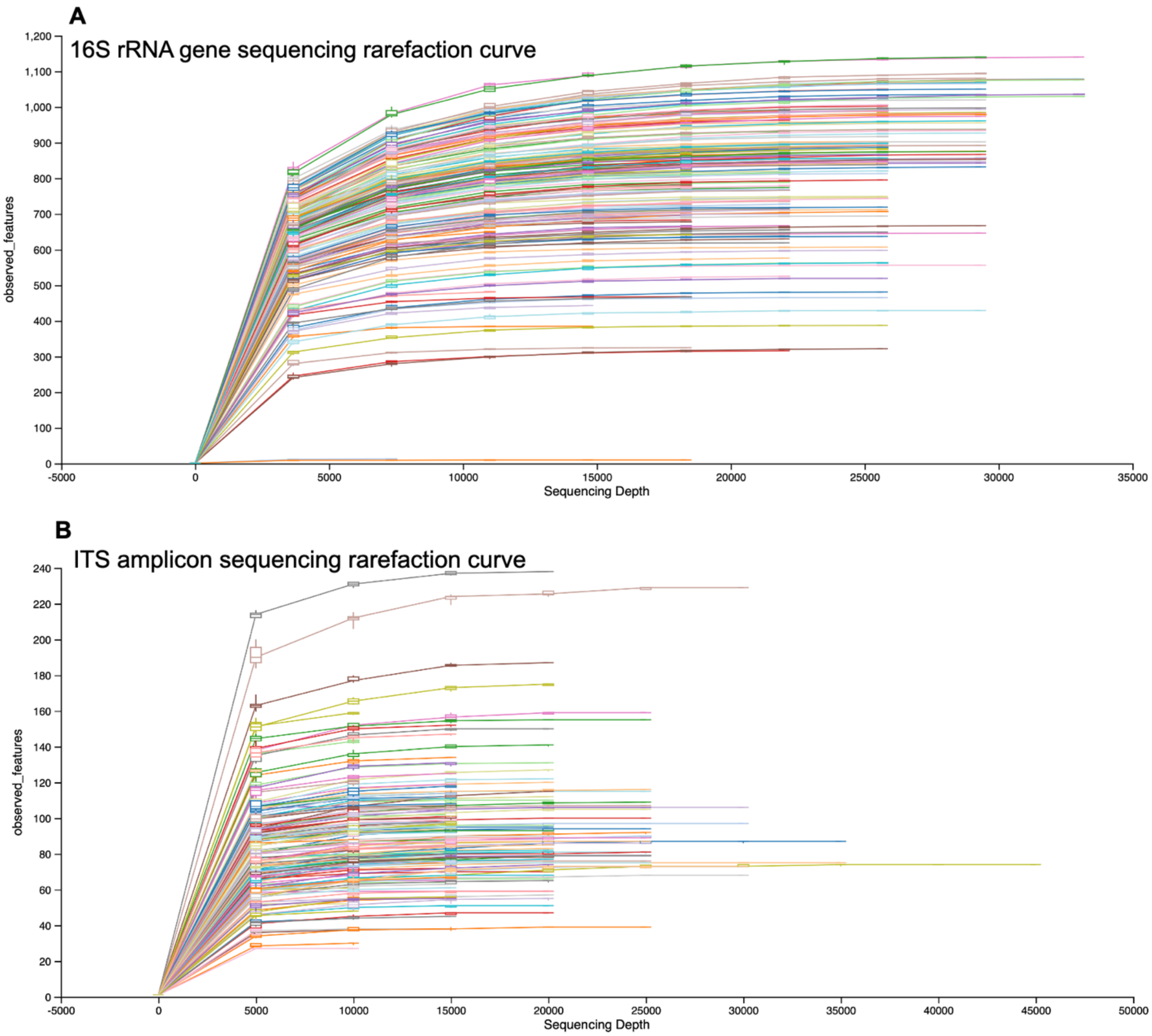

Fig. S1. Amplicon sequencing rarefaction curves. Rarefaction curves of 16S rRNA gene (A) and ITS amplicon (B) sequencing data, generating using QIIME2 diversity plug-in *alpha-rarefaction* function to assess whether samples were sufficiently sequenced to compare alpha diversity. Each line represents an individual sample.

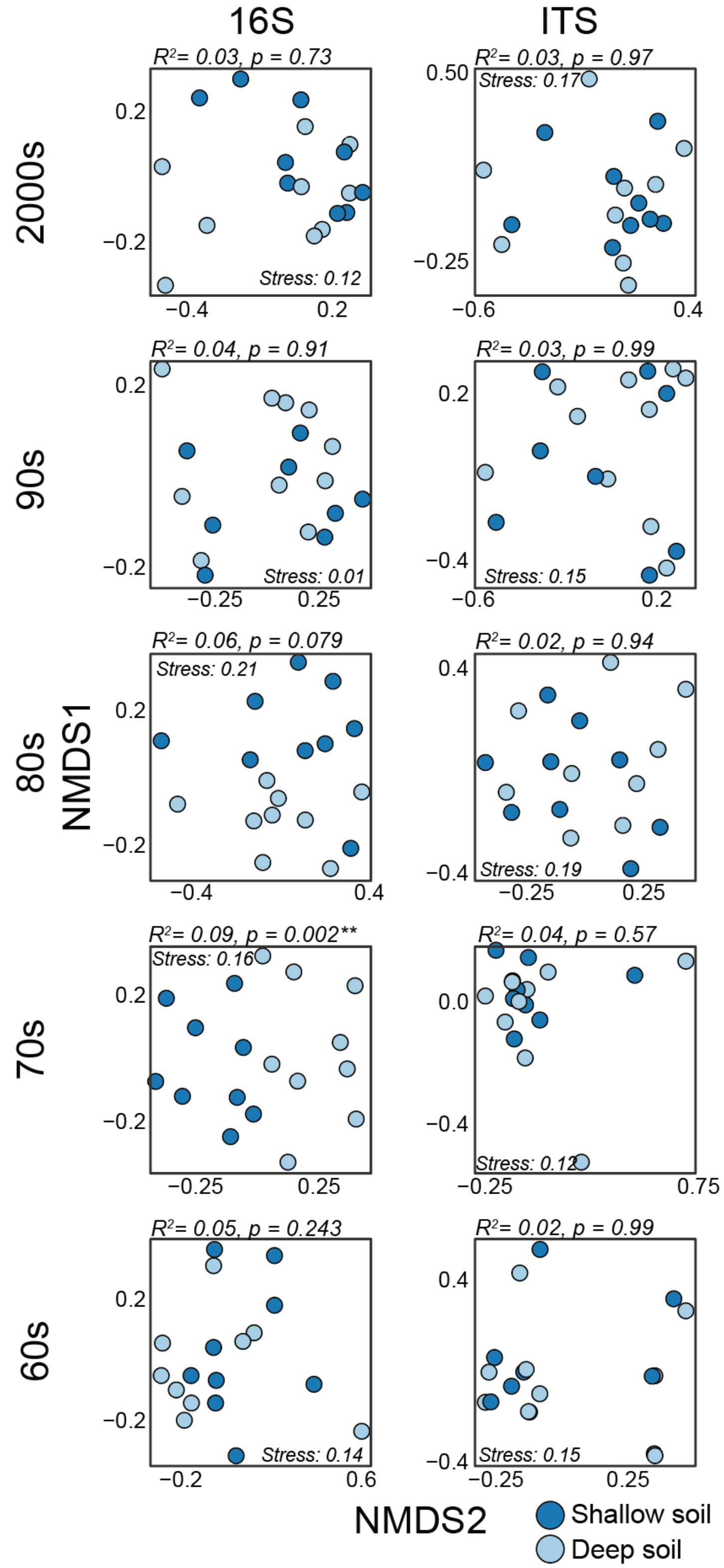

Fig. S2. Bacterial and fungal communities do not differ by sampling depth. Non-metric multidimensional scaling (NMDS) ordinations of bacterial (left) and fungal (right) communities from burned shallow (dark blue) and deep (light blue) samples across all burn decades. The only communities that are significantly different (assessed using PERMANOVA, stats on each NMDS plot) between depths are the 70s bacterial communities. The similarity between depths is likely due to the deep penetration of heat into the soil column when burning the high fuel loads used on burn piles. Because of this, the shallow and deep soil samples are combined for all analyses presented here.

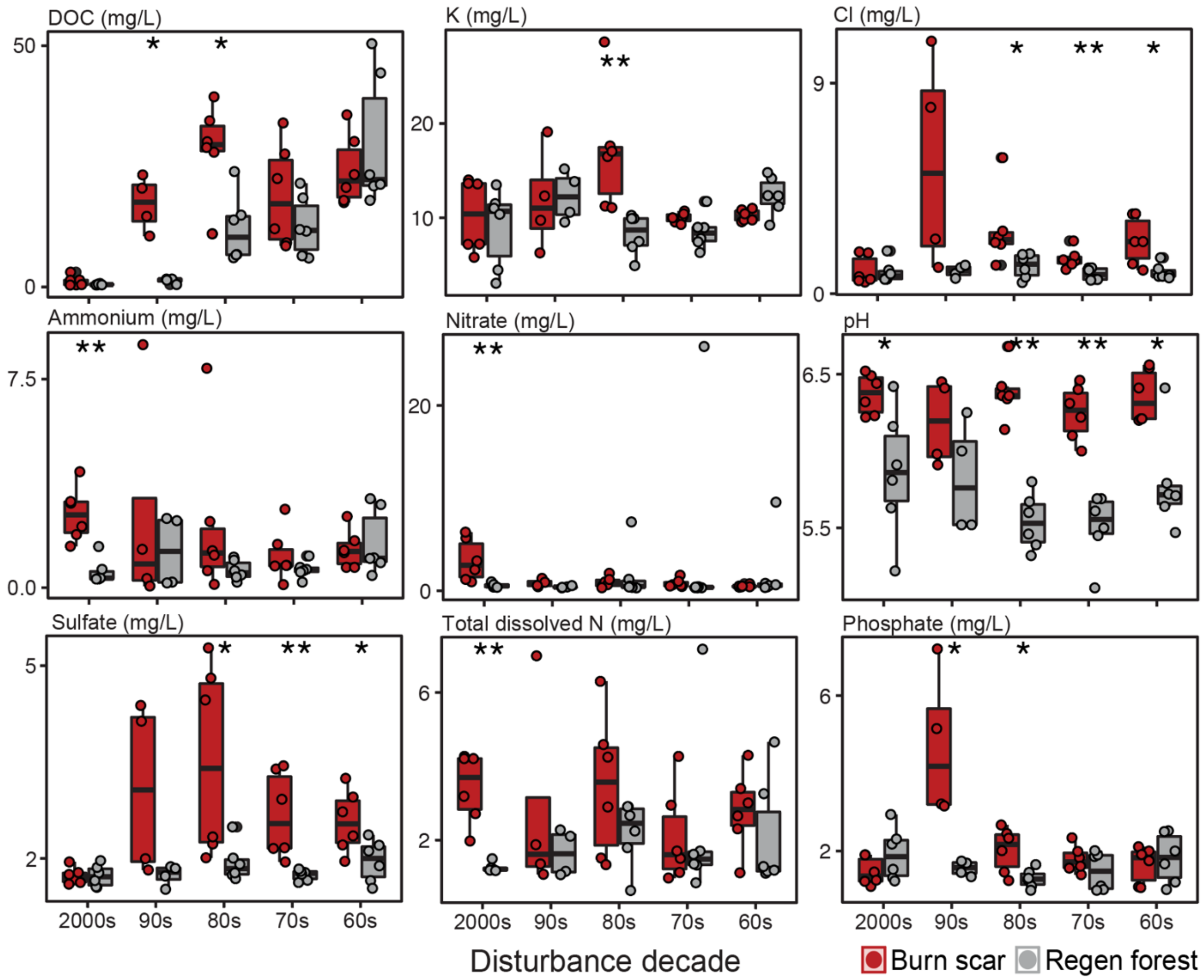

Fig. S3. Soil chemistry data. Soil chemistry data from water extracts of a subset of samples (n = 60). Points indicate individual sample data. The lower and upper hinges of the boxplots represent the 25th and 75th percentile and the middle line is the median. The upper whisker extends to the median plus 1.5x interquartile range and the lower whisker extends to the median minus 1.5x interquartile range. Significant differences between burn scar and regen forest samples indicated with asterisks as indicated by Wilcoxon rank-sum test. *p<0.05, **p<0.01.

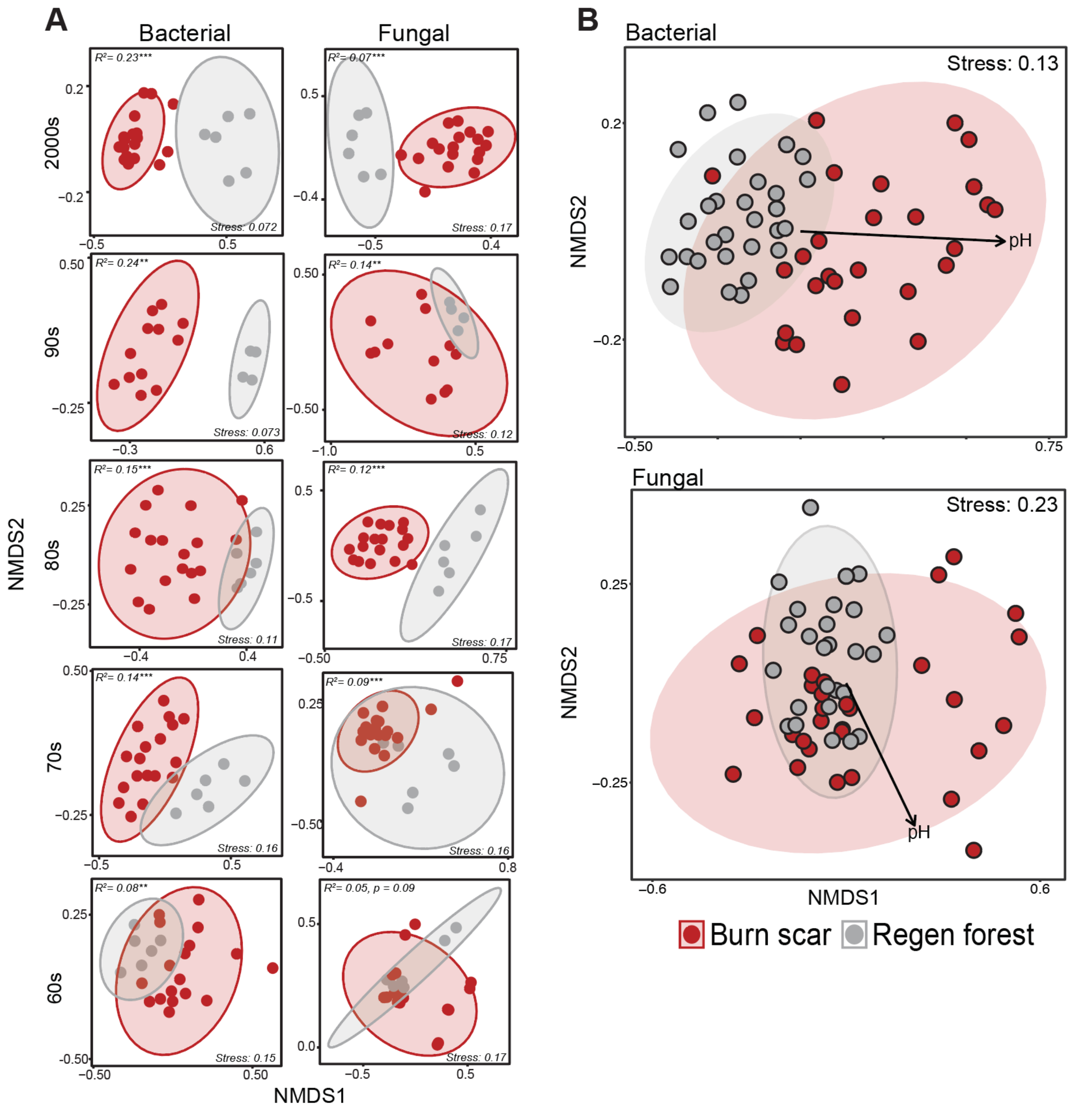

Fig. S4. Compositional dynamics of soil bacterial and fungal communities. (A) Non-metric multidimensional scaling (NMDS) ordinations of bacterial and fungal communities over time since disturbance, with corresponding PERMANOVA values indicating whether burn scar and regen forest sample communities are significantly different from one another. **(B)** NMDS of all bacterial and fungal communities with overlaying vectors indicating which measured soil chemistry variables significantly drive compositional shifts (via *envfit*; $p < 0.05$, corrected for multiple testing using Bonferroni's correction). Vector length indicates the magnitude of effect, and proximity of points to vectors indicates correlation. Note that, in Panel B, only the subset of samples from which there is corresponding soil chemistry data are included. All ellipses show ninety-five confidence interval for each treatment.

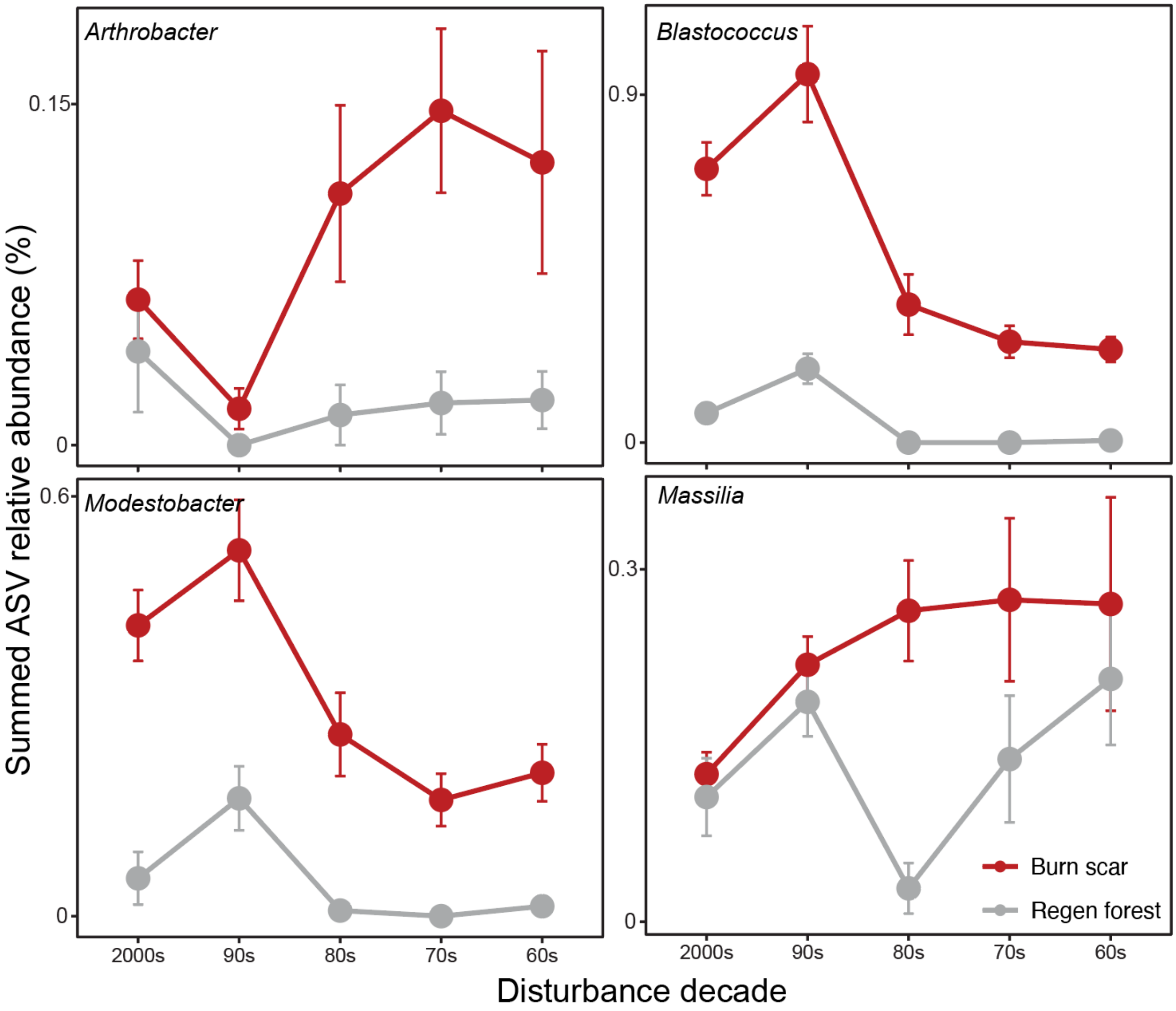

Fig. S5. Putative pyrophilous bacterial taxa generally enriched in burn scar soils. Summed ASV relative abundance of putative pyrophilous taxa, including the Actinobacteria genera *Arthrobacter (n* = 9 ASVs), *Blastococcus (n* = 22 ASVs), and *Modestobacter (n* = 9 ASVs), and the Proteobacteria genera *Massilia (n* = 22 ASVs). Error bars indicate the standard error of the mean.

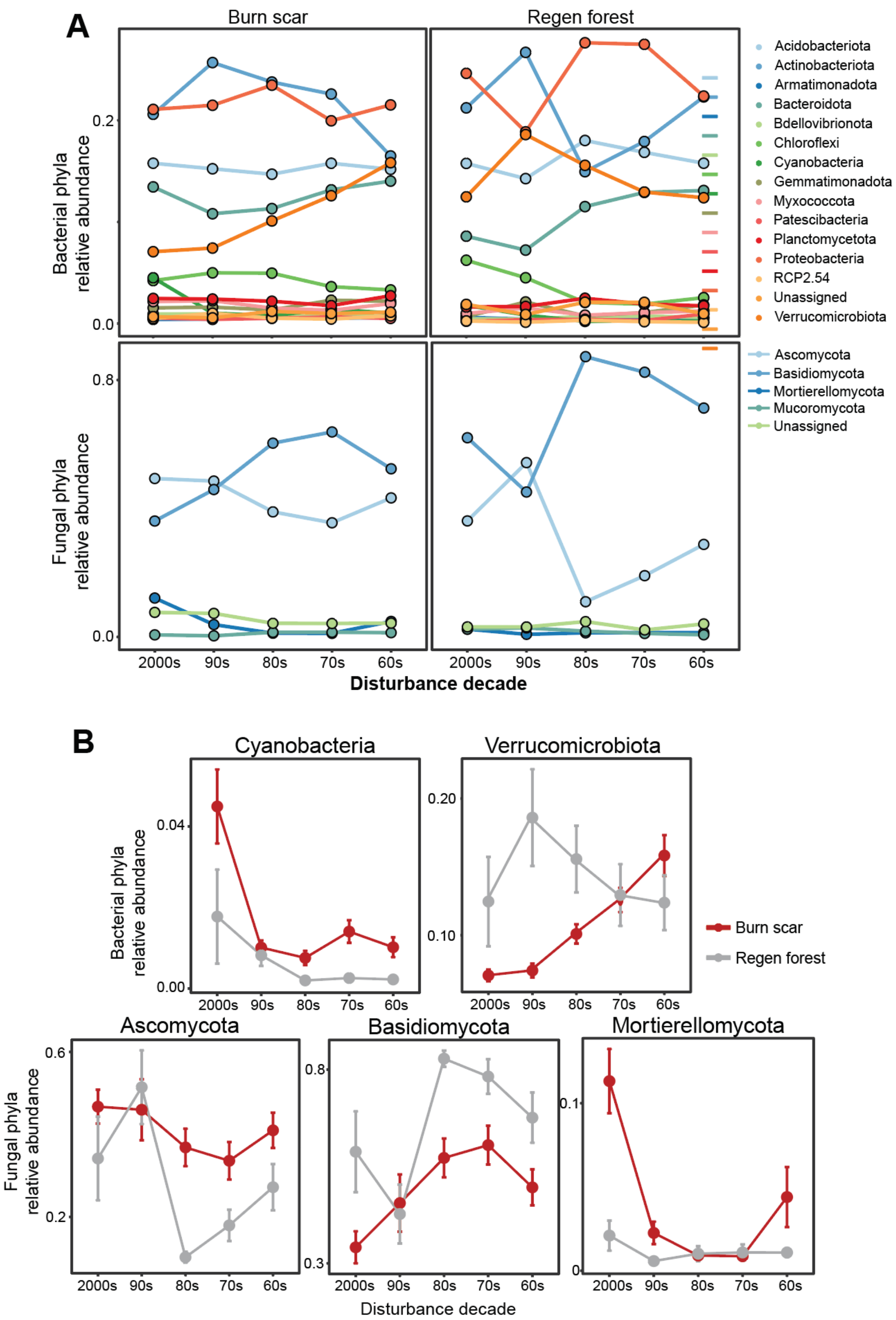

Fig. S6. Bacterial and fungal compositional shifts. (A) Dominant bacterial (top) and fungal (bottom) phyla in burn scar and regen forest soils over time. Phyla with average relative abundances <0.5% across samples were discarded. (**B**) Bacterial and fungal phyla of interest relative abundance over time between burn scar and regen forest soils. Error bars indicate the standard error of the mean.

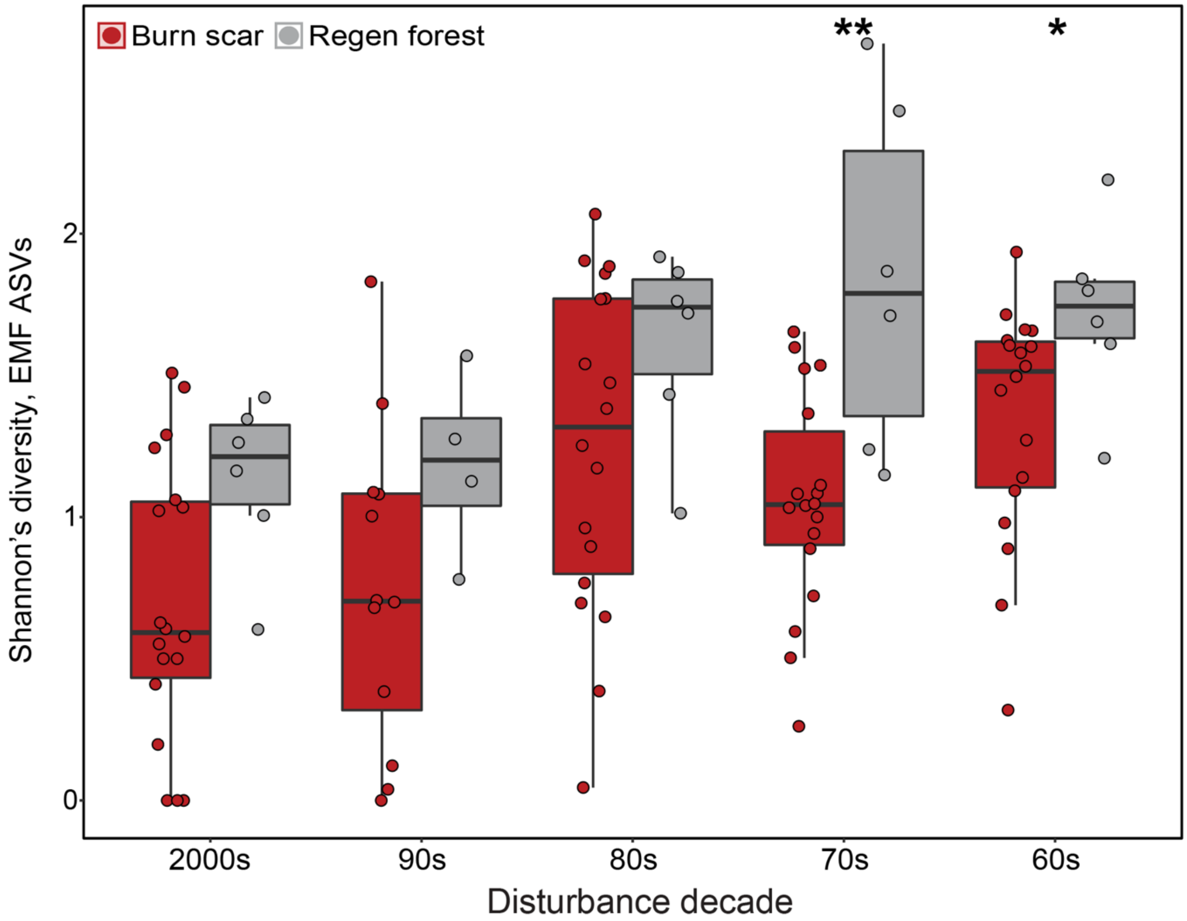

Fig. S7. EMF depleted following disturbance. Shannon's diversity of EMF communities in burn scar and regen forest soils. The lower and upper hinges of the boxplots represent the 25th and 75th percentile and the middle line is the median. The upper whisker extends to the median plus 1.5x interquartile range and the lower whisker extends to the median minus 1.5x interquartile range. Significant differences between burn scar and regen forest samples indicated with asterisks as indicated by Wilcoxon rank-sum test. *$p<0.05$, **$p<0.01$.

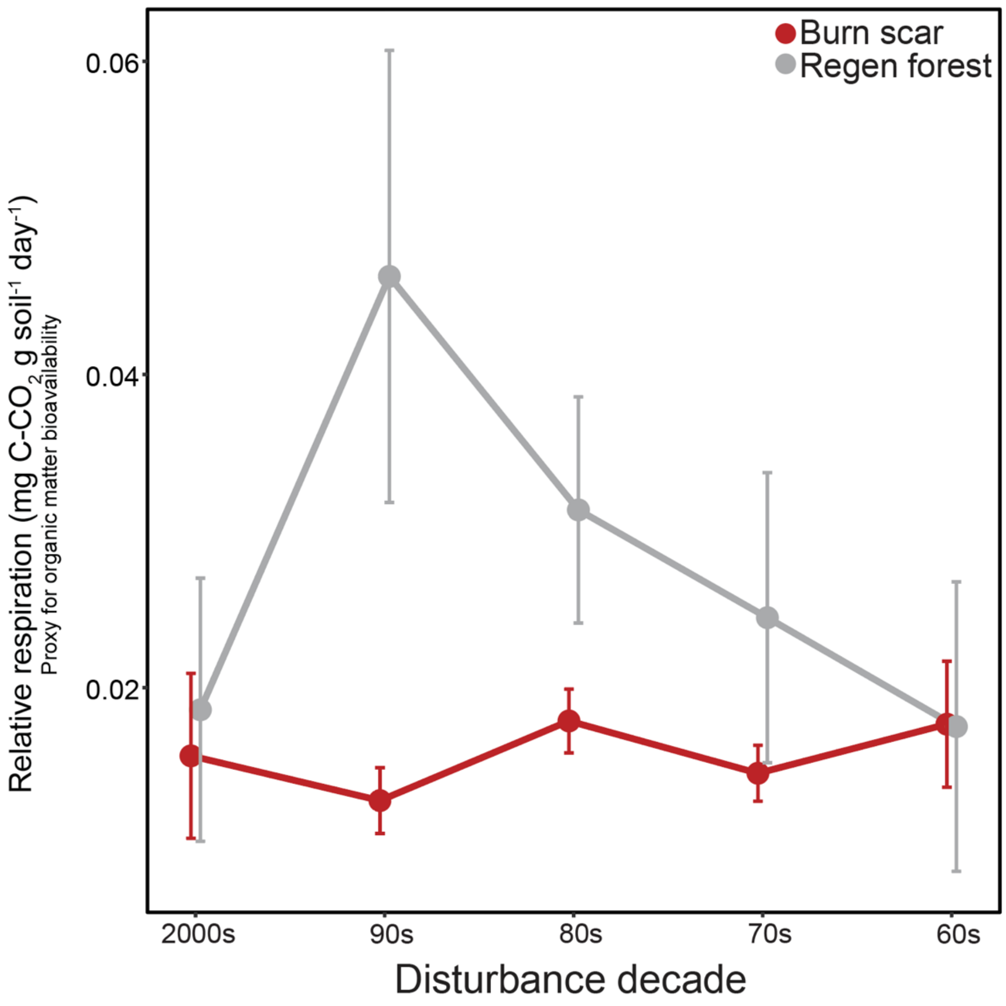

Fig. S8. Soil respiration generally depleted in burn scars. Average soil respiration, from laboratory incubations, of both burn scar and regen forest soils. Error bars represent the standard error of the mean.

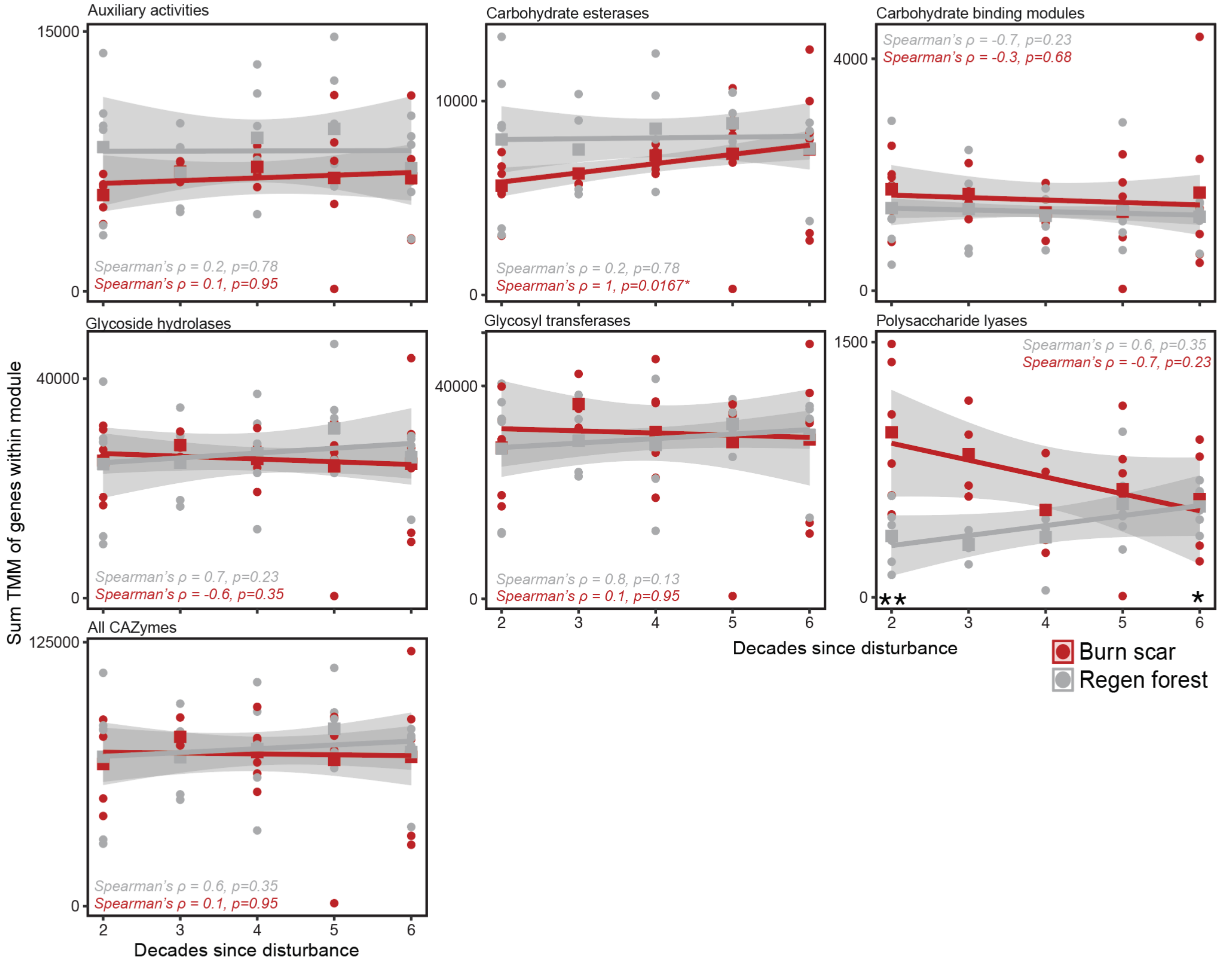

Fig. S9. CAZyme dynamics differ between treatment. Sum TMM of CAZyme gene categories in different treatments. Circles show individual samples and squares show averages from these samples. Linear regression was run as a function of average sum TMM of genes within each treatment by decades since disturbance, and Spearman's rho values are reported. Shaded area shows 95% confidence interval of linear model. Significant differences between burn scar and regen forest samples within each decade are shown with asterisks as indicated by Wilcoxon rank-sum test. *p<0.05, **p<0.01.

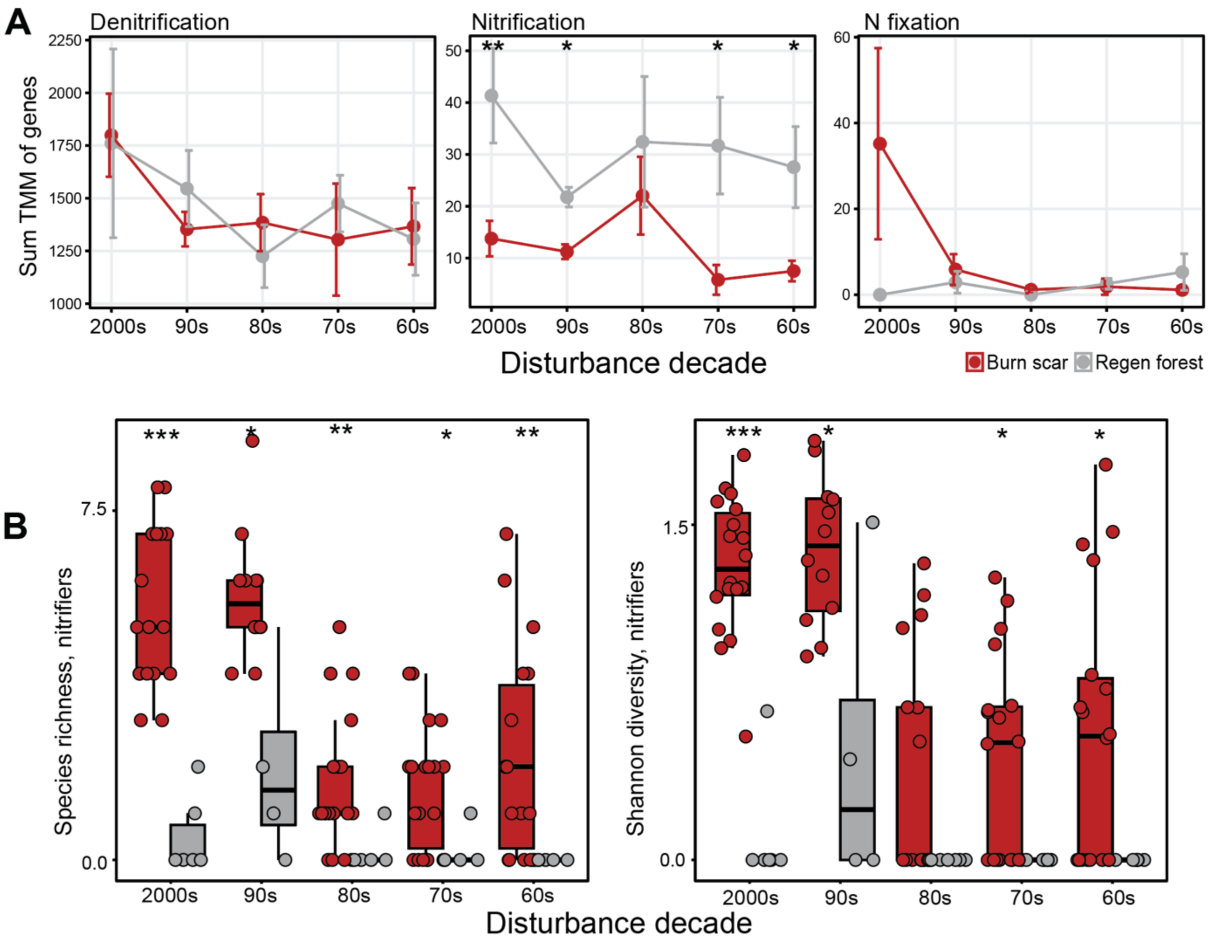

Fig. S10. Nitrogen functionality over time. (**A**) Average sum TMM of genes for denitrification, nitrification, and N fixation over time. Error bars represent the standard error of the mean. (**B**) Diversity of putative nitrifiers from 16S rRNA gene sequencing data. Significant differences between burn scar and regen forest samples within each decade are shown with asterisks as indicated by Wilcoxon rank-sum test. **p*<0.05, ***p*<0.01, ****p*<0.001.

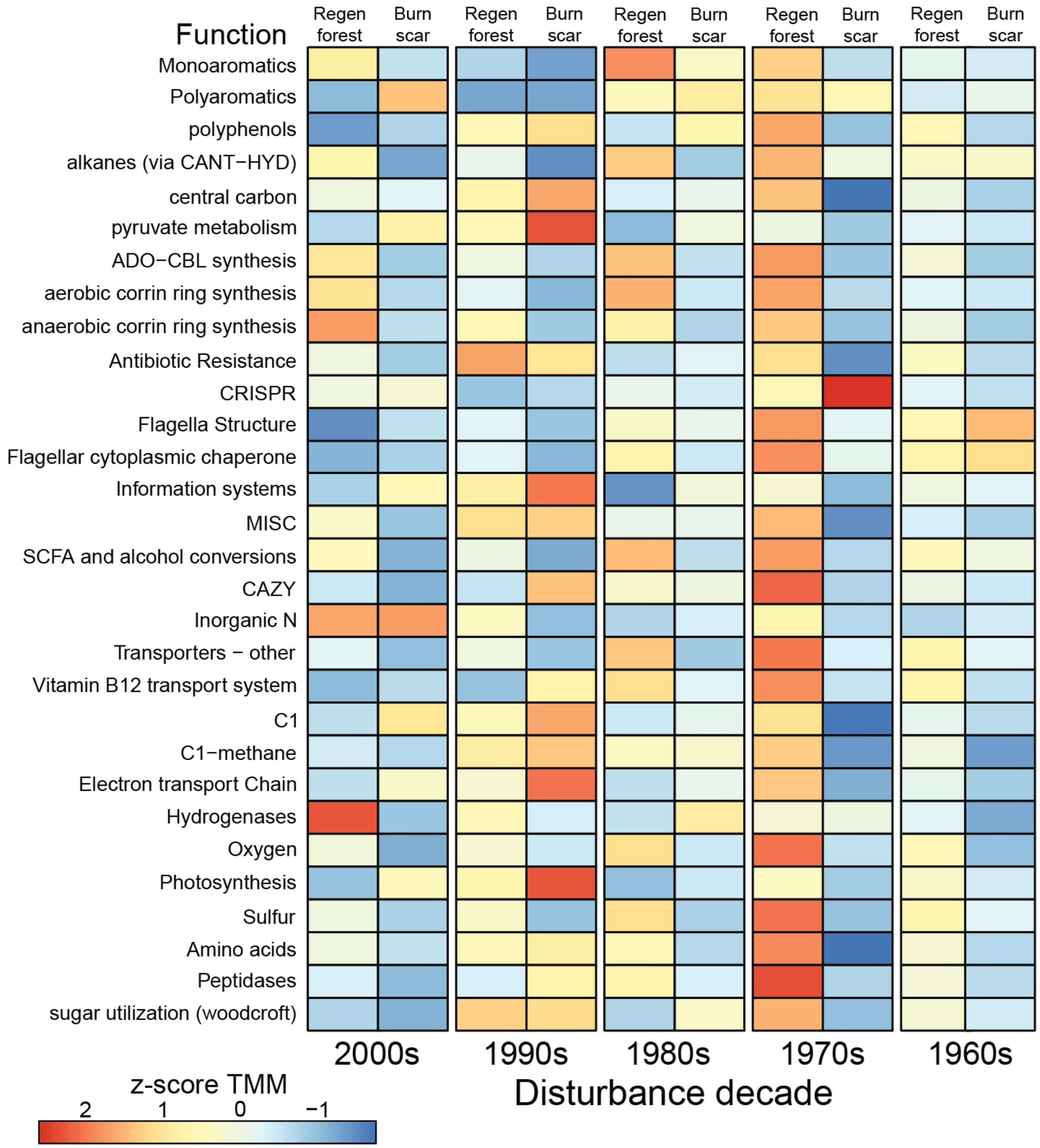

Fig. S11. Broad functional potential shifts in soil microbiome. Z-score of the average TMM of genes within broad functional groups across treatments and disturbance decade. Genes grouped into broad functional groups by DRAM-assigned function 'header' and CANT-HYD annotation of genes for degrading alkanes, monoaromatics (added to genes annotated by DRAM), and polyaromatics (added to genes annotated by DRAM). Z-score was calculated within each individual function.

a

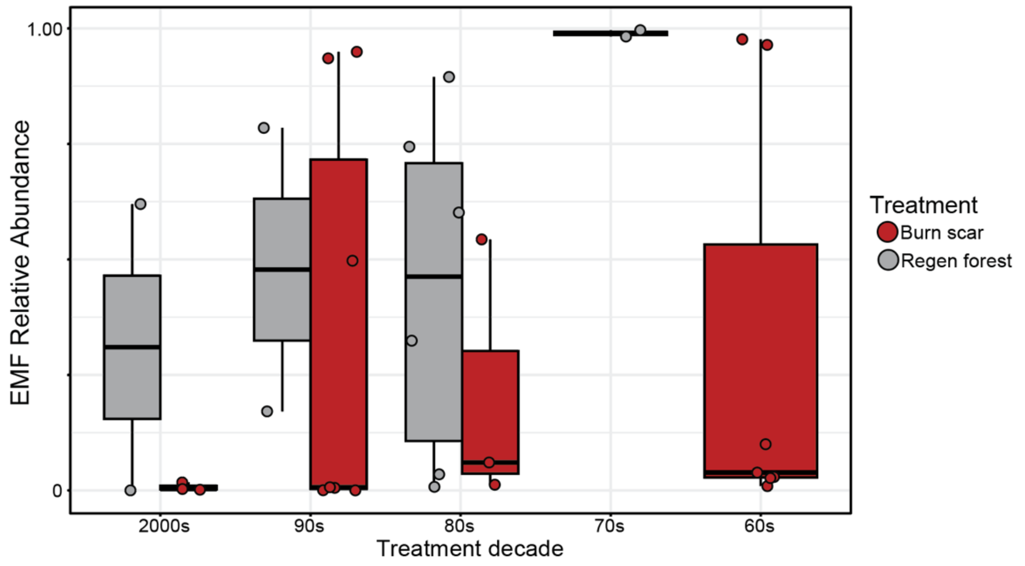

b Samples sequenced (#)

Decade	Burn scar	Regen
2000s	3	2
1990s	7	2
1980s	3	6
1970s	0	2
1960s	7	0

n = 32

c

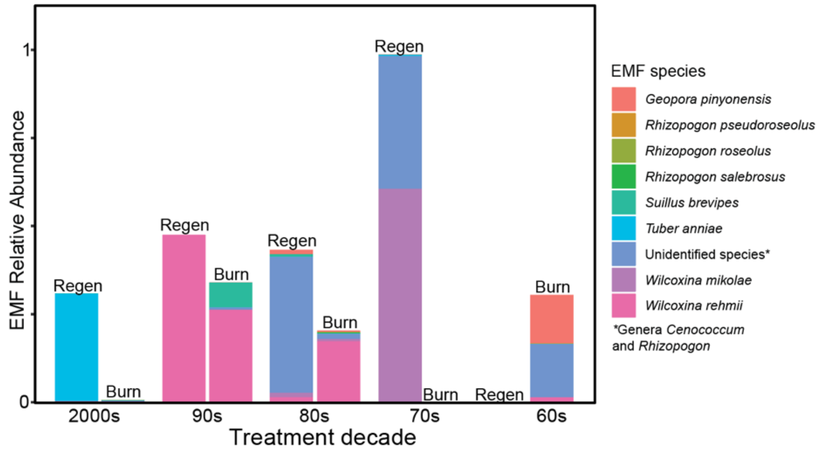

Fig. S12. Greenhouse bioassay experiments indicate poor EMF colonization of pine seedlings across treatments. (a) EMF relative abundance from DNA extracted from root nodules of pine seedlings grown in soils collected from burn scars and regen forest sites across the chronosequence. (b) Total number of pine seedling root tips used for DNA sequencing. DNA was extracted from root nodules of pine seedlings that had EMF root colonization. (c) EMF relative abundance, colored by EMF species, of root nodules. See **Supplementary Data 1 (Sheet C)** for relevant data.

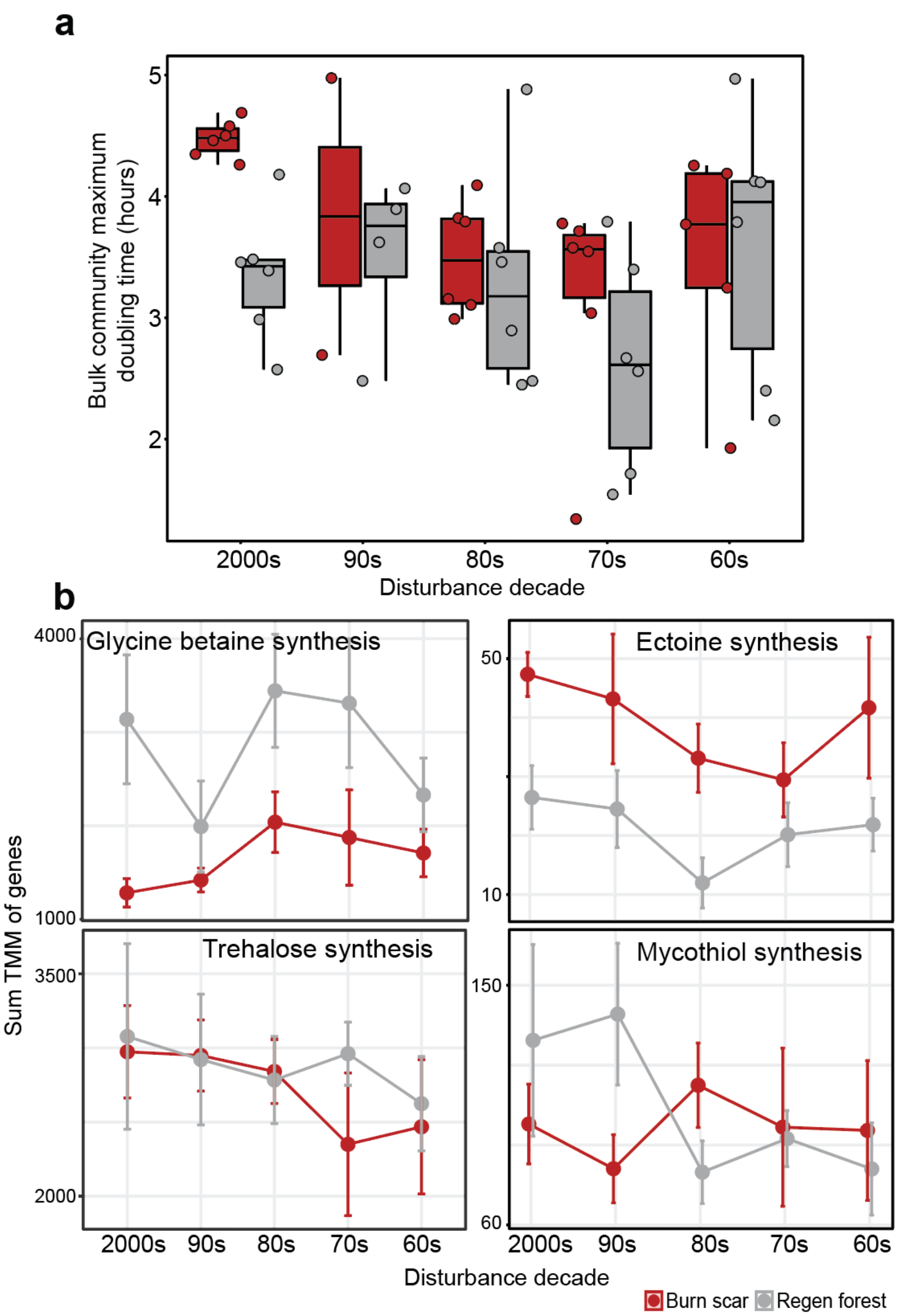

Fig. S13. Pyrophilous traits differ over multidecadal scales. (a) Bulk community maximum doubling time (via gRodon, metagenome mode) across samples. **(b)** Summed normalized abundance of genes for synthesis of different stress protectants.

Decade	*Treatment*	*# Samples*	*# Samples utilized for metagenomic sequencing*
**2000s**	*Burn scar*	18	6
	Regen forest	6	6
	Rhizosphere	9	N/A
**1990s**	*Burn scar*	12	4
	Regen forest	4	4
	Rhizosphere	6	N/A
**1980s**	*Burn scar*	18	6
	Regen forest	6	6
	Rhizosphere	9	N/A
**1970s**	*Burn scar*	18	6
	Regen forest	6	6
	Rhizosphere	9	N/A
**1960s**	*Burn scar*	18	6
	Regen forest	6	6
	Rhizosphere	9	N/A

Table S1. Number of soil samples collected per treatment and number of samples utilized for metagenomics. 154 total soil samples collected.

Decade	*Treatment*	*# Differentially abundant genes*	*Total # in decade*
**2000s**	Burn scar	218938	
	Regen forest	214588	433526
**1990s**	Burn scar	86997	
	Regen forest	57924	144921
**1980s**	Burn scar	194786	
	Regen forest	58192	252978
**1970s**	Burn scar	90073	
	Regen forest	116275	206348
**1960s**	Burn scar	39531	
	Regen forest	41410	80941

Table S2. Number of differentially abundant genes (calculated via DESeq2; $p<0.01$) within each treatment across decades. Calculated within-decade.

Supplementary Data 1. All sample metadata, associated chemistry, and greenhouse bioassay experiment data.

Supplementary Data 2. Ecological modeling (i.e., β-nearest taxon index, βNTI, and Raup-Crick, RC_{BC}) analyses.

Supplementary Data 3. All metagenomics mapping information including sequencing depth, % reads assembled or binned, and number of MAGs used from each metagenome. This data file also includes all MAG information (completeness, contamination, taxonomy, etc.) for MAGs generated from this set of metagenomes.

Supplementary Data 4. All additional supplemental annotations used along with DRAM and gene annotations and associated coverage data associated with C and inorganic N cycling and functions presented in Fig. 4. This data file also includes the summed average geTMM of all broad functional categories across treatments.

SI References

1. Dove NC, Taş N, Hart SC. Ecological and genomic responses of soil microbiomes to high-severity wildfire: linking community assembly to functional potential. The ISME Journal. 2022 Jul 16;16(7):1853–63.

2. Nelson AR, Narrowe AB, Rhoades CC, Fegel TS, Daly RA, Roth HK, et al. Wildfire-dependent changes in soil microbiome diversity and function. Nature Microbiology. 2022;1–12.

3. Whitman T, Whitman E, Woolet J, Flannigan MD, Thompson DK, Parisien MA. Soil bacterial and fungal response to wildfires in the Canadian boreal forest across a burn severity gradient. Soil Biology and Biochemistry. 2019 Nov 1;138:107571.

4. Whitman T, Woolet J, Sikora M, Johnson DB, Whitman, Ellen. Resilience in soil bacterial communities of the boreal forest from one to five years after wildfire across a severity gradient | Elsevier Enhanced Reader. Soil Biology and Biochemistry. 2022;(172):108755.

5. Weissman JL, Hou S, Fuhrman JA. Estimating maximal microbial growth rates from cultures, metagenomes, and single cells via codon usage patterns. PNAS. 2020;118(12):1–10.

6. Newton GL, Buchmeier N, Fahey RC. Biosynthesis and Functions of Mycothiol, the Unique Protective Thiol of Actinobacteria. Microbiology and Molecular Biology Reviews. 2008 Sep;72(3):471–94.

7. Richter AA, Mais CN, Czech L, Geyer K, Hoeppner A, Smits SHJ, et al. Biosynthesis of the Stress-Protectant and Chemical Chaperon Ectoine: Biochemistry of the Transaminase EctB. Frontiers in Microbiology. 2019 Dec 10;10(December):1–20.

8. Rhoades CC, Fornwalt PJ. Pile burning creates a fifty-year legacy of openings in regenerating lodgepole pine forests in Colorado. Forest Ecology and Management. 2015 Jan;336:203–9.

9. Faria SR, De La Rosa JM, Knicker H, González-Pérez JA, Villaverde J, Keizer JJ. Wildfire-induced alterations of topsoil organic matter and their recovery in Mediterranean eucalypt stands detected with biogeochemical markers. European Journal of Soil Science. 2015 Jul;66(4):699–713.
